# Mapping the genetic landscape of the age at onset and severity of eating disorder symptoms

**DOI:** 10.1192/bjp.2026.10763

**Published:** 2026-10-07

**Authors:** Helena L. Davies, Abigail R. ter Kuile, Zain-Ul-Abideen Ahmad, Sang Hyuck Lee, Rujia Wang, Jessica Mundy, Jonathan Coleman, Carlotta Sampels, Saakshi Kakar, Emily Kelly, Chelsea Mika Malouf, Gursharan Kalsi, Moritz Herle, Gerome Breen, Christopher Hübel

**Affiliations:** 1 Social, Genetic and Developmental Psychiatry Centre, Institute of Psychiatry, Psychology and Neuroscience, King’s College London, London, UK; 2 UK National Institute for Health and Social Care Research (NIHR) Biomedical Research Centre for Mental Health, South London and Maudsley Hospital, London, UK; 3 Department of Clinical, Educational, and Health Psychology, University College London, London, United Kingdom; 4 Department of Psychology, Humboldt-Universität zu Berlin, Berlin, Germany; 5 National Centre for Register-based Research, Aarhus University, Aarhus, Denmark; 6 Clinic for Child and Adolescent Psychiatry, Psychotherapy and Psychosomatics, German Red Cross Hospitals Westend, Berlin, Germany

## Abstract

**Background:**

Eating disorder treatment is most successful when delivered at first onset, underscoring the importance of early intervention. Genetic factors may contribute to earlier onset, but molecular genetic research into symptom onset and severity remains limited.

**Aims:**

We investigated (1) age at onset of behavioural symptoms and low weight, including sex differences, (2) the SNP-based heritability and cross-trait polygenic associations of symptoms, onset, and severity.

**Method:**

Participants (n = 24,973) of genetically-derived European ancestry were from the NIHR BioResource, including the Genetic Links to Anxiety and Depression Study, Eating Disorders Genetics Initiative UK, and COVID-19 Psychiatry and Neurological Genetics Study. Symptoms were assessed with the ED100K questionnaire. We described sex-stratified onset of binge eating, low weight, self-induced vomiting, laxatives, diuretics, excessive exercise, and fasting. SNP-based heritability was estimated using GCTA-GREML. We tested associations with 26 polygenic scores (PGS), which were calculated using SBayesRC.

**Results:**

Symptom median onset ranged from 16-20 years: vomiting (16), fasting and excessive exercise (17), binge eating (18), laxatives (19), diuretics (20), and low weight (20). Males were disproportionately likely to report adult onset. In females, behavioural symptoms and low weight showed substantial heritability (SNP-based *h*^2^ = 0.31–0.78), but onset and cognitive symptoms appeared to be less heritable. PGS analyses identified distinct pathways: in females, higher childhood obesity PGS was linked to earlier binge eating onset (~7 months), and higher educational attainment PGS to earlier low weight onset (~6 months).

**Conclusions:**

Eating disorder symptoms begin in adulthood as frequently as adolescence, challenging stereotypes and underscoring investment needed in adult services, particularly for men. While symptoms were strongly heritable, onset and cognitive symptoms showed limited genetic contribution. Symptom-level PGS analyses highlight distinct biological and developmental pathways. Larger sample sizes and more accurate phenotyping are needed.

## Introduction

Eating disorders affect at least ~1.4 million people in the UK (1). The most-studied are: anorexia nervosa, which presents as severe weight loss (BMI <18.5) due to restriction or excessive exercise with a perception of fatness despite low weight (2); bulimia nervosa, which involves binge eating followed by compensatory behaviours such as self-induced vomiting; and binge-eating disorder, which presents as binge eating without compensation (2). Treatment outcomes for eating disorders are poor (3–6), but interventions are most successful when delivered at first onset (7). Thus, early intervention is important (8).

### Age at onset of eating disorders and treatment in the UK

Early identification should target the transition from adolescence into emerging adulthood as this is when eating disorders typically begin. A meta-analysis (k=192) summarised retrospectively reported median age at symptom onset (7): anorexia nervosa was 16 years, bulimia nervosa was 18 years, and binge-eating disorder was 20 years (9–11). No UK samples were included, which we address with our study. In the UK, authorities require treatment to be delivered in age-appropriate settings (12) and patients turning 18 years old move to adult services. This transition lacks structure and is complicated by changes in treatment, social environment, and family structure (12). A 2019 National Health Service (NHS) England Guidance called for a seamless transition pathway (13). Moreover, eating disorder symptoms differ by onset; compared to an adolescent onset (13–20 years), children (<13 years) report less binge eating, purging, or excessive exercise for weight loss (14,15) and lose weight at a faster pace (14). Adolescent onset of anorexia nervosa shows lower death rates and better long-term outcomes than later onset (16), whilst onset in childhood shows worse outcomes (17,18).

### Genetics of eating disorders and their age at onset

Genetic risk may influence age at onset, as eating disorder symptoms are heritable. Twin-based heritability estimates range from 0-66% for anorexia-nervosa-specific symptoms, 43-72% for bulimia-nervosa-specific symptoms, and 41% for binge eating in the absence of compensatory behaviours (a core symptom specific to binge-eating disorder; Table S1 including references). Evidence shows that early onset may reflect greater genetic susceptibility (19–24). For example, higher PGGs for schizophrenia, major depression, autism spectrum disorder were significantly associated with a younger age at onset of bipolar disorder (24). The lack of an association between the bipolar disorder PGS and bipolar disorder age at onset may be due to a lack of predictive power resulting from a less well-powered discovery GWAS. The Brainstorm Consortium reported a modest negative correlation between average age of onset and heritability across 22 brain disorders, including anorexia nervosa (25). For instance, Tourette’s syndrome showed 20% heritability with an average onset of 7 years whilst major depressive disorder showed 13% heritability with an average onset of 32 years (25). However, there were a number of outliers, e.g., schizophrenia showed a SNP-based heritability of 26% with an average age of disorder onset of 21 years. Adult-onset ADHD is associated with a lower ADHD polygenic score than childhood-onset, suggesting distinct subtypes, with the former being more environmentally influenced, less consistent, or milder (26). Sex-specific genetics may also play a role in onset: early-onset schizophrenia in females may reflect greater genetic susceptibility, whilst males may accumulate more environmental risks (19). Such findings emphasise how genetic risk interacts with early environment and sex-at-birth.

Genome-wide complex trait analysis via genomic-relatedness-based restricted maximum-likelihood (GCTA-GREML) of the age of diagnosis of anorexia nervosa showed a SNP-based heritability of 1%, 21% for early-onset (diagnosis <13 years, assuming lifetime prevalence 0.3%), and 15% for typical-onset (>13 years, assuming lifetime prevalence of 4%, Table S1), suggesting that earlier onset may be driven in part by genetics (27). Early onset showed a negative genetic correlation with age at menarche, and Mendelian randomisation suggested a causal role of younger age at menarche on early-onset anorexia nervosa (27). These findings indicate distinct sub-phenotypes influenced by puberty timing. An age at menarche polygenic score also correlated negatively with binge-eating disorder (28) and positively with adolescent persistent thinness (29), suggesting broader relevance for eating disorders and weight phenotypes beyond anorexia nervosa.

### Aims

As of 2026, the genetics of eating disorder symptoms and their clinical course, including onset and severity, have been minimally investigated. Therefore, we performed a two-fold investigation: 1) phenotypic analysis exploring the age at onset of low weight and behavioural symptoms - including binge eating, self-induced vomiting, diuretic and laxative use, fasting, and excessive exercise - in a large UK sample; 2) a genetic analysis to assess the SNP-based heritability and cross-trait polygenic risk score association of a) behavioural eating disorder symptoms and low weight and b) their age at onset, and c) cognitive symptoms and d) their severity.

## Methods

### Sample

Participants were from the National Institute for Health and Care Research (NIHR) BioResource (*n* = 70,442) who participated in the Genetic Links to Anxiety and Depression (GLAD) Study (30) (*n* = 46,802), the Eating Disorders Genetics Initiative (31) (EDGI UK; *n* = 6,393), or other physical health and general population cohorts (*n* = 17,247) whose participants took part in the COVID-19 Psychiatry and Neurological Genetics (COPING) Study (Table S2). The COPING Study provided complementary data for GLAD and EDGI participants to their sign-up survey (32).

We excluded 45,469 participants missing sex (measured via self-reported assigned sex at birth; *n* = 1,826), age (*n* = 1,474), or eating disorder symptoms (*n* = 41,321). The ED100K was optional in GLAD, which explains the high missingness. We excluded 1,322 participants with inflammatory bowel syndrome (IBS) or inflammatory bowel disease (IBD) at random to match the general population’s illness prevalence of 1.2% and between 10-20%, respectively (33). We checked prevalence in cases and controls separately; 11.5% of cases had IBD which we matched in the control sample. Oversampling for these conditions could confound the analyses by introducing symptom and genetic effects related to IBD/IBS, making it harder to identify associations specific to eating disorders. 24,973 individuals reported at least one eating disorder symptom, whilst 16,846 had age at onset data. We excluded 337 participants with genetically-inferred non-European ancestry via principal components analysis. We removed genetic variants with minor allele frequency <1%, that were missing in >5% of participants, or that deviated from Hardy-Weinberg equilibrium (*p* < 10^-10^). We excluded participants with >5% missing variants, with undetermined genetic sex, or with high mean identity-by-descent to other participants (*n* = 409), leaving 15,559 individuals with both phenotypes and genetic data. For the PGS analysis, we removed UK Biobank participants (*n* = 1,132) to avoid sample overlap with discovery genome-wide association studies (GWASs) conducted within the UK Biobank.

### Ethical approval

The authors assert that all procedures contributing to this work comply with the ethical standards of the relevant national and institutional committees on human experimentation and with the Helsinki Declaration of 1975, as revised in 2013. The London - Fulham Research Ethics Committee approved the GLAD Study, 21.08.2018 (REC reference: 18/LO/1218) and EDGI UK on 29.07.2019 (REC reference: 19/LO/1254). The NIHR BioResource is approved as a Research Tissue Bank by the East of England - Cambridge Central Committee (REC reference: 17/EE/0025). The COPING study is approved by the South West - Central Bristol Research Ethics Committee on 27.04.2020 (REC reference: 20/SW/0078). All participants provided informed consent.

### Measures

#### Behavioural eating disorder symptoms and low weight

Participants reported binge eating, low weight, self-induced vomiting, laxative use, diuretic use, fasting, and excessive exercise via the ED100K (34) (Table S3). This questionnaire was included in the sign-up survey in EDGI UK and COPING and was optional in GLAD. We identified participants who reported a lifetime symptom and as a comparison, participants who who endorsed not having experienced that symptom and who were not recruited on the basis of having a psychiatric disorder (i.e., were not participants of the GLAD Study or EDGI).

Participants endorsing a symptom could report the age at onset (Table S3): for example, “*Roughly how old were you when you began having regular episodes of binge eating?*”. For binge eating, participants must endorse loss of control (*n*_excluded_ = 13). We removed implausible responses younger than 3 and older than 118 years (*n*_excluded_ = 257) and ages that exceeded the participant’s current age (*n*_excluded_ = 58).

#### Cognitive symptoms

Participants reported the severity of five cognitive symptoms on Likert scales: 1) self-worth being dependent on body shape or weight when at low weight and 2) when binge eating, 3) fear of weight gain at low weight, 4) feeling fat at low weight, and 5) feeling that body parts are larger than they are at low weight (Table S3). We generated binary (symptom present or absent) and continuous (severity scores) variables for all symptoms except ‘feeling that body parts are larger’, which we retained as a 3-point scale (“Not at all”, “Somewhat”, “Very much”). For the remaining continuous variables, we excluded participants who endorsed absence of the symptom, creating severity scales ranging from 4 to 6 points (depending on the symptom) that reflect the range in those with the symptom.

### Analysis

Paper has been preprinted (35). We used R version 4.1.2 (36).

### Retest-reliability of retrospective self-reported age at onset

A small sub-sample of participants completed the ED100K twice: first, optionally in the GLAD Study and second, mandatorily in the COPING study (*n* = 239 to 1,805 participants, depending on symptom). We assessed participants’ reliability of their retrospective reports by calculating the difference between both answers. For our analyses, we used participants’ most recent responses.

### Phenotypic analysis

#### Age at onset

We described combined and sex-stratified age at onset. We calculated percentages in onset categories by sex: (1) Childhood: ≤12 years, (2) Adolescence: 13–17 years, (3) Emerging adulthood (37): 18–25 years, and (4) Adulthood: ≥26 years. We additionally explored the role of sex and gender by regressing behavioural symptoms and low weight and their age at onset on sex (male/female) and gender (man/woman/transgender).

### Genetic analysis

#### SNP-based heritability

Using GCTA-GREML (38), we estimated SNP-based heritabilities in females only (insufficient male cases, *n*_max_ = 665). Population prevalences for liability-scale estimation of behavioural symptoms and low weight were estimated from the part of the sample not ascertained for a psychiatric disorder. We used our sample’s prevalence estimates for the cognitive symptoms, as they are likely to best reflect the population prevalence given that they exist only in cases (e.g., feeling fat *at low weight*). We estimated SNP-based *h*^*2*^ for 1) quantitative and 2) binary age at onset, split into adult (≥18 years) and child/adolescent onset (<18 years). Quantitative traits were right-skewed and thus square-root transformed to approximate normality (Figure S1). We assessed cognitive symptom severity using linear models, assuming an underlying latent normal distribution.

Analyses were adjusted for genotyping batches and ten genetic principal components. As age is correlated with differences in reporting onset between the GLAD and COPING studies (r = 0.14–0.28), we controlled for age in an additional analysis of onset.

#### Cross-trait polygenic associations

We created psychiatric, anthropometric, social, behavioural, somatic, and personality polygenic risk scores (PGS; for discovery GWASs, see Table S4) using SBayesRC (39). Using linear and logistic (binary and ordinal) regressions, we used these PGSs as exposure variables and explored their associations with the following outcomes: 1) behavioural symptoms and low weight, 2) their age at onset, 3) cognitive symptoms and 4) their severity in both females and males (where sample size allowed). PGSs were standardised. We used the same adjustment variables as above. We corrected for multiple testing using FDR with a 0.01 threshold.

## Results

### Sample descriptives

Participants had an average age of 45 years (SD=18), were majority female (73.5%), most self-reported their race as white (96.2%), and 18% had a Master’s degree or above (for full details, see Table S5).

### Retest-reliability of retrospective self-reported age at onset

Binge eating showed the largest discrepancies in reported age at onset between first and second reporting in the GLAD and COPING studies, with 12.0% of the differences exceeding 10 years compared with 3.1% to 8.5% for the other eating disorder symptoms (Table S6; Figure S2). The mean and median differences ranged from 2 to 4.4 years and 1 to 2 years, respectively. However, the correlations between both assessment time points ranged from 0.69 to 0.85 for all symptoms, indicating acceptable retest-reliability (Table S6).

### Phenotypic analysis

#### Age at onset

Self-induced vomiting had the youngest mean onset at 18 years in females (Table 1; Table S7; Figure 1A). Fasting and laxative use in males had the oldest mean onset at 33 and 35 years. Males consistently showed an older mean age at onset than females across all symptoms; for example, the mean age at onset of binge eating was 20 in females but 25 years in males. To assess the influence of reporting bias on our outcome distributions, we excluded participants whose discrepancy between two reported ages of symptom onset exceeded 5 years, showing minimal difference in the outcome distributions (Table S8).

Females more frequently reported adolescent onset (35–53%) than males (12–34%), whilst males more frequently reported adult onset (25–71%) than females (9–32%) (Figure 1B; Table S9). Further, more females than males reported onset in childhood of excessive exercise (16% vs. 2%) and fasting (16% vs. 4%).

Compared with men, all other gender groups (non-binary, transgender, and women) reported significantly earlier ages at onset, with differences ranging from approximately 4 to 18 years (Table S10). For symptom presence, the sample included sufficient numbers of women and men without the symptoms to estimate odds ratios; women had significantly greater odds of all symptoms than men (ORs between 6.71 and 23.16). Compared to being male, being female was also associated with significantly greater odds of all symptoms (ORs ranging from 6.7 to 22.05; Table S10) and with younger ages at onset (ranging from 4 to 12 years younger).

### Genetic analysis

#### SNP-based heritability

**Figure 2:**
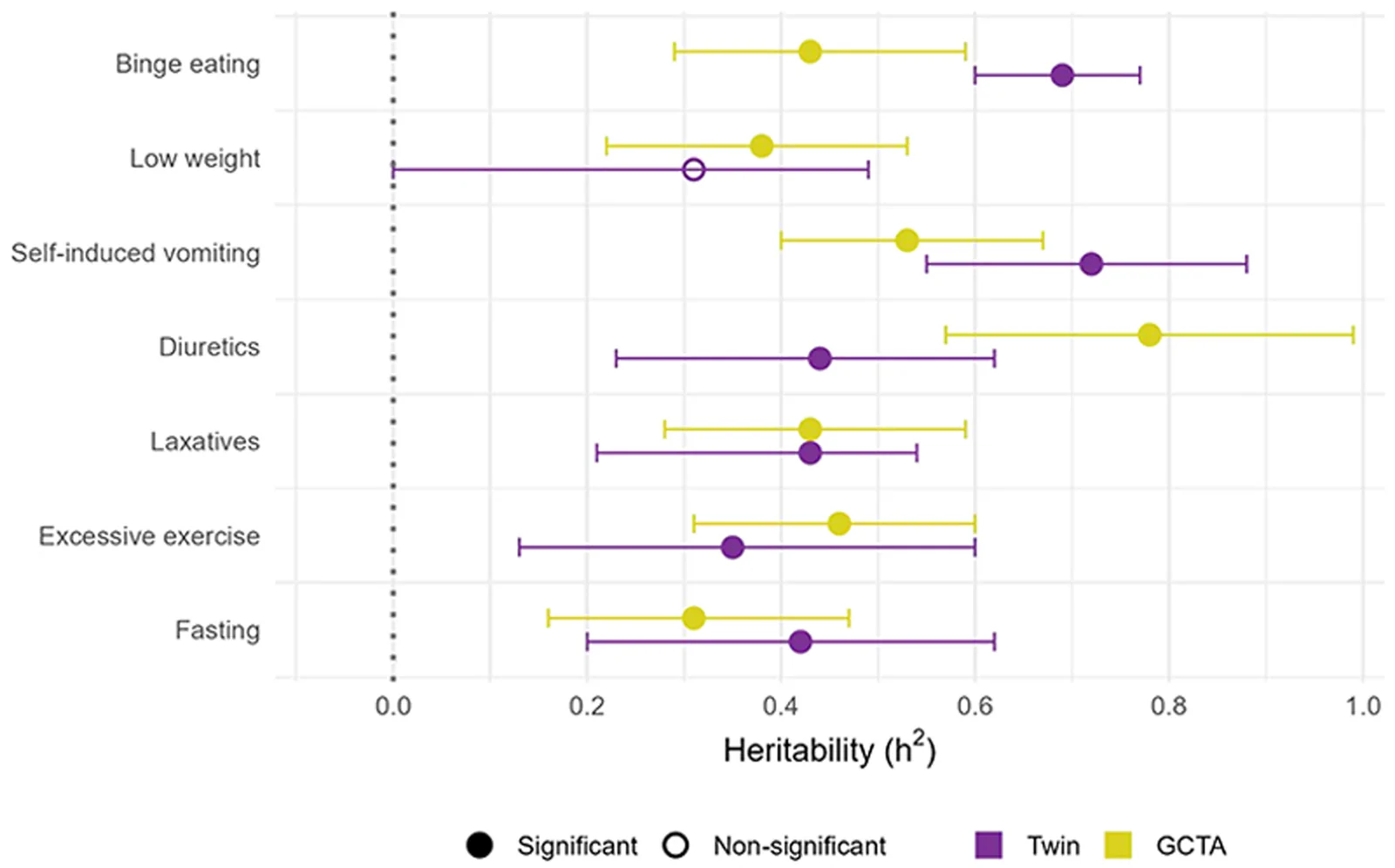
GCTA-GREML and twin heritability estimates in females of lifetime presence of binge eating, low weight, self-induced vomiting, diuretics, laxatives, excessive exercise, fasting. *Note*. References for twin heritability estimates are in Table S1. Covariates included ten principal components and genotyping batches. Prevalence of lifetime symptoms for liability-scale conversion is calculated from the National BioResource sample (i.e., participants not recruited on the basis of having a psychiatric disorder). P-values are adjusted via False Discovery Rate.

We detected significant SNP-based heritability for all behavioural symptoms and low weight (Figure 2; Table S11; Table S12 shows a range of liability-scale estimations). Diuretic use had the highest heritability estimate (0.78 [SE = 0.10]) whilst fasting was the least heritable with (0.31 [SE = 0.08]).

Neither the continuous nor the binary age at onset phenotypes showed a significant heritable component after correcting for multiple testing (Figure S3; Table S11) or adjusting for age (Table S13). GCTA-GREML power calculations (40) indicated that our smallest sample (n_diuretic use_ = 979) translated to 9% power to detect a heritability of 0.20 and 80% power to detect a heritability of 0.90. Our largest sample (n_fasting_ = 5,668) had 95% power to detect a heritability >0.20, suggesting that the maximum heritability of this symptom’s continuous age at onset phenotype is <0.20. With this phenotype, we had 15% power to detect a heritability of 0.05, as was found for depression by Harder et al (2022).

We did not identify heritable components for cognitive symptoms or their severity at the current sample size (Figure S3; Table S11). Power calculations indicated that our largest sample sizes (*n*_symptom_ = 4,849 and *n*_severity_ = 4,273) were 100% powered to detect a heritability of 0.50 and 0.30, respectively, placing an upper limit on the likely heritabilities of these phenotypes.

#### Cross-trait polygenic associations

**Figure 3:**
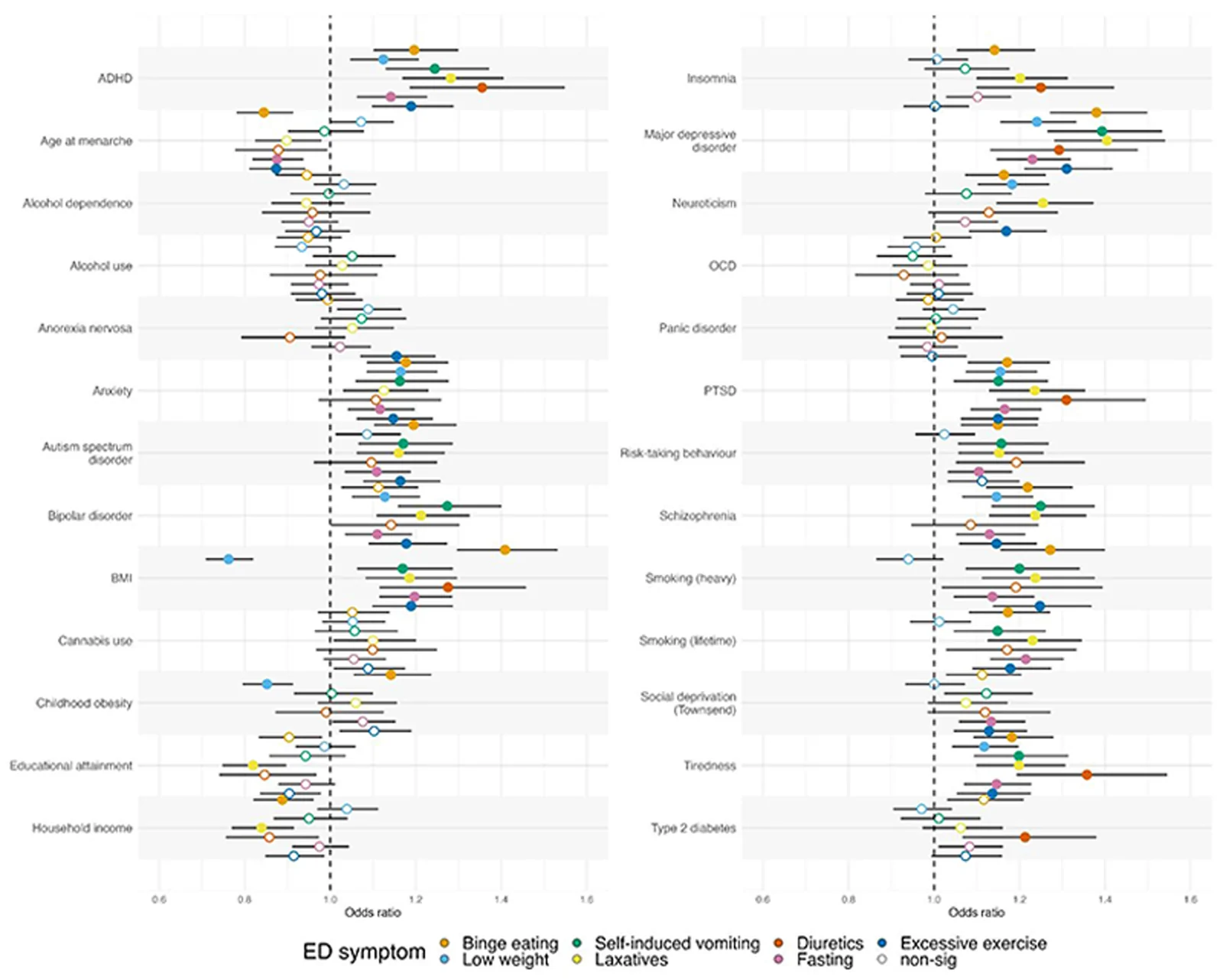
Anthropometric, psychiatric, social, behavioural, somatic, and personality polygenic risk score associations with lifetime behavioural eating disorder symptoms and low weight in females.

We observed multiple consistent significant associations across the behavioural symptoms and low weight in females (Figure 3; Table S14) and males (Figure S4; Table S15). For example, the ADHD PGS was associated with greater odds of all symptoms in females (ORs = 1.12–1.36, *q*s<0.001) and with binge eating and excessive exercise in males (OR=1.35, 95% CI 1.17, 1.56, *q*=0.001; OR=1.19, 95% CI 1.07, 1.32, *q*=0.01, respectively). Other PGSs showed diverging associations. For example, for each standard deviation (SD) increase in the childhood obesity PGS in females, the odds of reporting low weight were lower by 0.15 (95% CI 0.8, 0.91, *q*<0.001) but 0.14 higher for reporting binge eating (95% CI 1.05, 1.24, *q*=0.003), and showed no significant associations with any other symptom.

Three PGSs were negatively associated with age at onset in females (Table S16) and none in males (Table S17). In females, higher childhood obesity PGS was associated with a younger onset of binge eating (β = -0.58, 95% CI -0.83, -0.32, *q*=0.002); with each SD increase in the PGS corresponding to, on average, a ~7 months earlier onset. Similarly, each SD higher educational attainment PGS was associated with an onset of low weight ~6 months earlier (β = -0.54, 95% CI -0.81, -0.27, *q*=0.007) whilst a one SD higher schizophrenia PGS was associated with an earlier onset of fasting by ~7 months (β = -0.53, 95% CI -0.81, -0.26, *q*=0.007).

We identified some significant PGS associations with cognitive symptoms and their severity in females (Tables S18 and S19) and none in males (Tables S20 and S21). In females, being afraid of weight gain was associated with anorexia nervosa PGS (OR=1.18, 95% CI 1.09, 1.28, *q*=0.001), BMI PGS (OR=1.25, 95% CI 1.15, 1.36, *q*<0.001) and childhood obesity PGS (OR=1.25, 95% CI 1.16, 1.35, *q*<0.001). Feeling fat at low weight was also associated with BMI PGS (OR=1.26, 95% CI 1.18, 1.36), *q*<0.001) and childhood obesity PGS (OR=1.18, 95% CI 1.10, 1.26, *q*<0.001). Further, severity of feeling fat at low weight was positively associated with ADHD PGS (OR=1.12, 95% CI 1.05, 1.20, *q*=0.006) and BMI PGS (OR=1.18, 95% CI 1.10, 1.26, *q*<0.001).

BMI PGS was also associated with greater severity of self-worth being dependent on body shape/weight at low weight (OR=1.16, 95% CI 1.09, 1.23, *q*<0.001) and feeling one’s body is larger than it is at low weight (OR=1.12, 95% CI 1.06, 1.20, *q*=0.004). Childhood obesity PGS was also associated with this latter symptom severity (OR=1.14, 95% CI 1.07, 1.21, *q*=0.001). Educational attainment PGS was associated with lower severity of self-worth being dependent on body shape/weight at low weight (OR=0.88, 95% CI 0.83, 0.94, q=0.001) and when binge eating (OR=0.89, 95% CI 0.84, 0.94, *q*=0.002). Finally, for each SD higher lifetime smoking PGS, the odds of being in a group that reports greater severity of self-worth being contingent on body weight/shape at low weight versus all groups reporting less severity combined was 0.13 odds higher, assuming proportional odds across thresholds (OR=1.13, 95% CI 1.06, 1.20, *q*=0.002).

## Discussion

### Phenotypic analysis: Age at onset

We extended international research by examining ages at onset for specific eating disorder symptoms in a UK sample. Median onset was 17 years for both fasting and excessive exercise, 16-20 years for purging behaviours (vomiting, laxatives, diuretics); and 18 and 20 years for binge eating and low weight. Our UK findings align with international estimates that eating disorders typically emerge between adolescence and early adulthood (7). Despite this existing research, there is a stereotype that eating disorders are primarily adolescent conditions. Our findings challenge this by showing onset is equally common in adulthood.

Our findings suggest that limited funding in adult services may disproportionately disadvantage men with eating disorders. Across all symptoms, a higher proportion of males than females reported adult onset (>18 years): binge eating (71% vs. 55%), low weight (81% vs. 57%), vomiting (57% vs. 34%), laxatives (75% vs. 48%), diuretics (81% vs. 53%), excessive exercise (80% vs. 40%), and fasting (82% vs. 42%). Male eating disorders remain under-recognised, under-researched, and shaped by female-centric diagnostic models (41), making inadequate adult service investment especially disadvantageous for men. These patterns may reflect genuine sex differences in onset, driven by pubertal timing, hormonal changes (42) or differential social and environmental risks. Alternatively, they may result from stereotypes framing eating disorders as female conditions (43), leading men to delay recognising symptoms and thus reporting later onset (44). Further research using longitudinal prospective research is needed to clarify these mechanisms.

We demonstrate the need for parity between child/adolescent and adult services in the UK. Historically, adult services have experienced greater constraints on funding and resources, with 55% of child/adolescent services reporting the discharge of patients at age 18 for not meeting the criteria for adult services (45). Stricter thresholds reflect limited resources. In 2014, the Children and Young Person’s community eating disorders services in England were allocated an additional £30 million (now ~£53 million) per year (46). Adult services saw no equivalent increase until the 2019 NHS Long Term Plan (47), which outlined investment in adult community eating disorder services to improve the continuity of care (48). Beyond different therapeutic approaches, transitions are hindered by poor communication, unclear guidelines, and disruption of established therapeutic relationships at a critical developmental stage (49–51). Our results support not only sustaining but expanding this investment.

### Genetic analysis: SNP-based heritability

We found that whilst each lifetime symptom has a significant genetic component (0.31–0.78), their age at onset appears less genetically influenced. Our null findings may reflect limited sample size; Harder et al. (2022) reported a 5.6% heritability for depression onset in 76,365 participants (21), while Ferentinos et al. (2015) found 17% in 3,468 participants using structured interviews. Interview-based phenotyping may yield more reliable data than surveys (52,53). Further, other quantitative phenotypes have demonstrated low heritability estimates, such as subjective well-being (h^2^ = 0.062 [SE = 0.005]), cigarettes per day (0.057 [0.013]), and extraversion (0.049 [0.008]) (25). GCTA-GREML heritability estimates are sensitive to measurement error (54), which may be greater in quantitative than binary phenotypes. For example, recalling whether symptoms began at 16 or at 17 is less reliable than before or after 30. Nonetheless, even binary onset phenotypes showed non-significant heritability. Watson et al. (2022) reported 1% heritability for age at diagnosis in anorexia nervosa, with 9,335 participants and mixed phenotyping methods, including clinical interviews. Thus, our null results may reflect both smaller sample size and measurement error.

We found no molecular genetic evidence for heritability of cognitive symptoms or their severity (e.g., fear of weight gain, self-worth contingent on body shape/weight), despite twin studies reporting moderate estimates (e.g., 23% for feeling fat at low weight) (55). As with age at onset, smaller genetic contributions may exist but remain undetectable in our sample. Larger meta-analyses are needed to clarify whether polygenic influences on these symptoms can be reliably identified.

Within the diathesis–stress framework, genetic and environmental factors interact to shape disease liability (56); our paper suggests that whilst eating disorder symptoms are heritable, common genetic variation may contribute little to onset timing or cognitive severity. Instead, environmental factors - such as early trauma (57), cultural pressure around thinness, urbanisation, industrialisation (58), consumerism, and changing gender roles (59) - along with non-common genetic factors, may better explain individual differences in these traits.

### Genetic analysis: Cross-trait polygenic associations

In females, we observed that a higher BMI and childhood obesity PGS were linked to lower odds of low weight. This supports prior findings that low-BMI PGS - combined with AN PGS - strongly shape BMI trajectories (60). We also provide the first evidence that greater childhood obesity genetic load is associated with earlier binge eating onset (~7 months) in females. Higher BMI PGS was positively associated with all behavioural symptoms in females and most in males, and many cognitive symptoms and their severity in females, consistent with prior associations between anthropometric PGSs and eating disorder symptoms such as body dissatisfaction and fasting (61).

The type 2 diabetes PGS was positively associated with diuretic use but not low weight in females, despite type 2 diabetes having a negative genetic correlation with AN (r_g_ = −0.22) (62). Likewise, the OCD PGS was unrelated to low weight, despite the known genetic overlap between OCD and AN (63) and body dysmorphic concerns (64), suggesting metabolic and psychiatric traits may differentially influence low weight and psychiatric symptoms of AN.

PGSs for ADHD, MDD, and PTSD in females showed positive associations across all behavioural symptoms and low weight, supporting shared psychiatric genetic risk (65). Many studies indicate a positive genetic correlation between body weight and ADHD (e.g. 66); whilst our finding of a positive association with binge eating supports this, our finding of a positive association with low weight does not. Nonetheless, we replicate links between earlier menarche and binge eating (28), and report, to our knowledge, novel associations between age at menarche PGS with fasting and excessive exercise. Mechanisms remain unclear: menarche and disordered eating are negatively genetically correlated (r_g_ = -0.18) (67); pubertal fat gain may heighten body dissatisfaction (68); and estrogen may moderate genetic effects on binge eating during puberty (69).

In females, the educational attainment PGS was linked to lower odds of laxative use and earlier onset of low weight, but not low weight itself. This latter finding contrasts with prior positive genetic correlations between years of schooling and both early- (r_g_ = 0.15) and typical-onset AN (r_g_ = 0.24) (27). Emerging evidence suggests the non-cognitive genetic component of education (e.g., curiosity, self-control) may drive this link (70). Our results highlight the value of symptom-level analyses for disentangling genetic pathways to eating disorder risk.

### Limitations

Our findings must be considered in light of multiple limitations. First, age at onset was measured by retrospective self-report, which is unreliable (34,71); in our data, 12% of binge eating onset estimates differed by ≥10 years. Question wording also varied. For example, that low weight showed the oldest onset - contrary to other studies (7) - may reflect its measurement as an *outcome* of other symptoms such as restrictive eating and weight loss behaviours. Compensatory behaviours’ first onset were recorded whilst binge eating was defined as clinically-relevant onset (“ߪ*regular episodes of binge eating*”). Whilst self-report enables efficient large-scale data collection, careful question design is important (see 72). Second, COPING data were collected throughout the COVID-19 pandemic, when environmental homogeneity may have inflated heritability. Third, our sample is largely white, female, and highly educated, with genetic analyses limited to European genetically-inferred ancestry; such selection can bias genotype-phenotype associations (73). Ongoing recruitment efforts in GLAD and EDGI UK aim to increase diversity, particularly among males and other genetic ancestries. Fourth, differences in the power of discovery GWASs may make direct comparison between PGSs difficult and have contributed to some results not reaching statistical significance, e.g., the association between the AN PGS and low weight.

### Future directions

Our work raises questions for future research. The establishment of twin-based heritability estimates for the age at onset of eating disorder symptoms would contextualise our SNP-based heritability estimates (27). Although incorporating age at onset can boost GWAS power, this relies on onset being heritable (21); larger samples and more precise phenotyping are needed to test the extent to which the age at which eating disorder symptoms start is heritable, and therefore the value of pursuing its addition to GWASs.

### Conclusion

To conclude, we show that eating disorders arise in adulthood as frequently as in childhood or adolescence, challenging the stereotype of primarily adolescent onset and carrying important clinical implications. The observed sex differences in age at onset may reflect true biological variation or reporting bias and require further study. While individual eating disorder symptoms are heritable, the timing of their onset and cognitive features appear only minimally influenced by common genetic variation.

## Supplementary Material

Supplementary Materials

## Figures and Tables

**Figure 1A:**
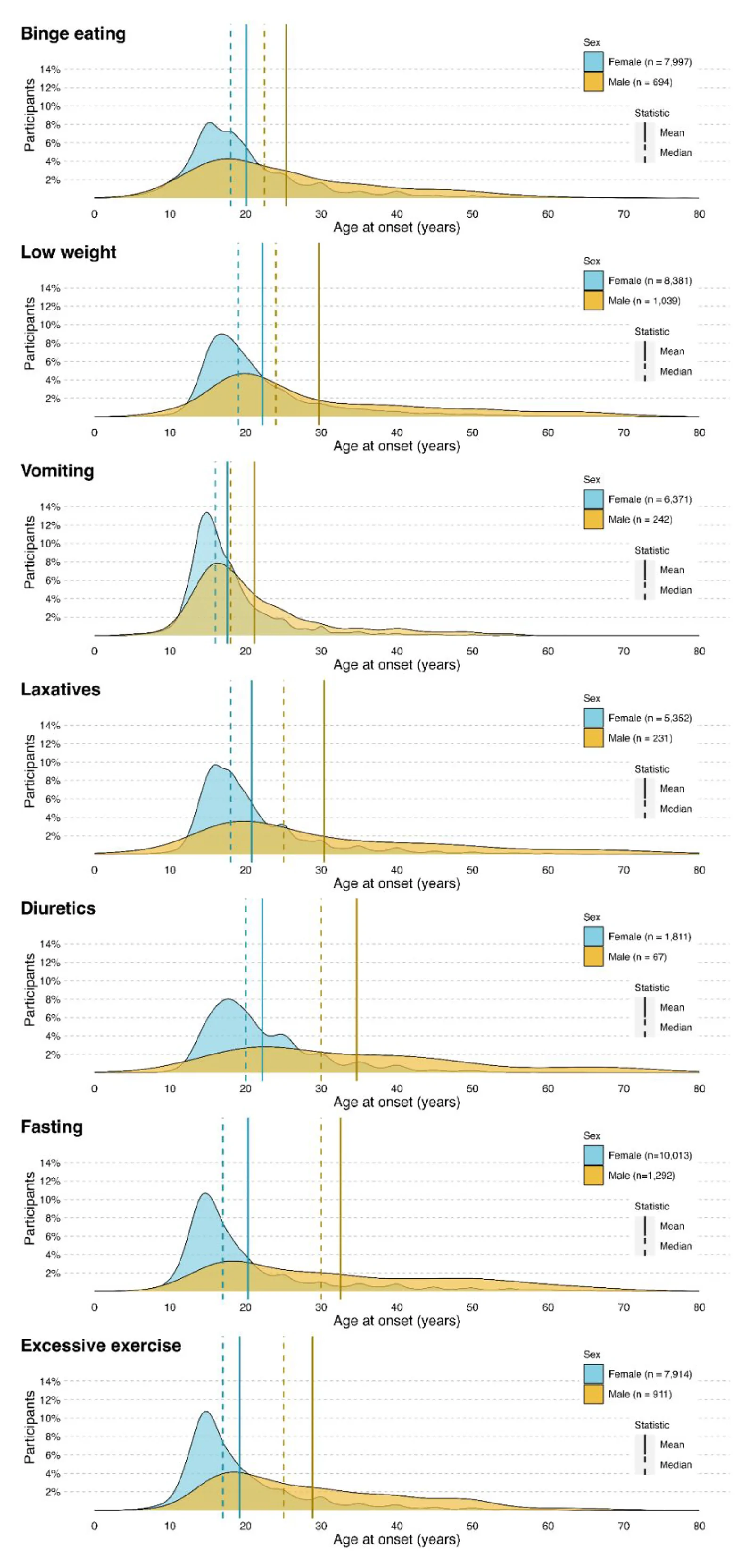
Sex-stratified age at onset of behavioural eating disorder symptoms and low weight.

**Figure 1B:**
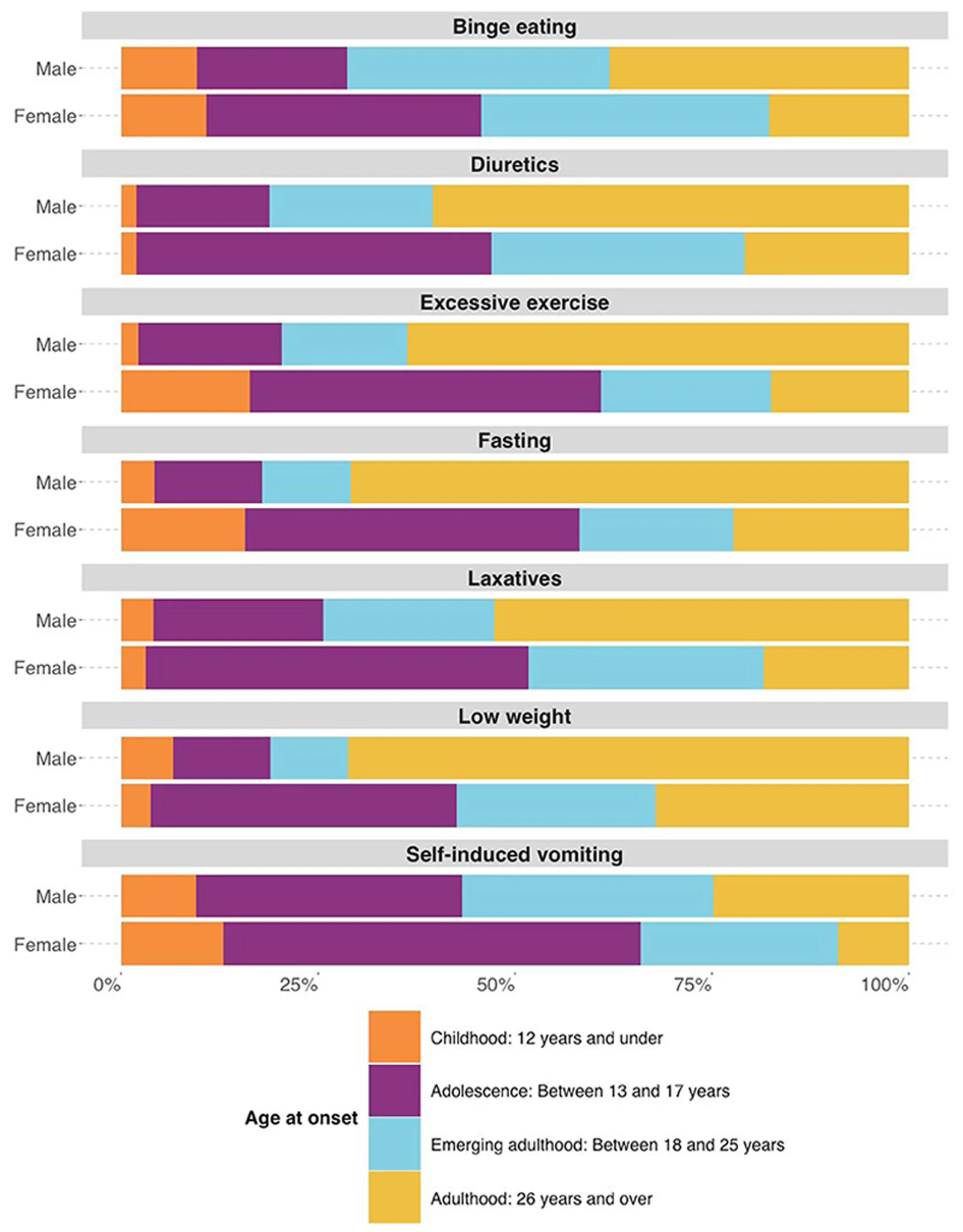
Sex-stratified age at onset split into four categories: 1) Childhood, 2) Adolescence, 3) Emerging adulthood, 4) Adulthood

**Table 1:** Mean age at onset of each phenotype in the entire sample, split by sex.

	Mean (SD)		
	Entire sample	Female	Male
Binge eating (n = 8,691)	20 (9)	20 (9)	25 (12)
Low weight (n = 9,420)	23 (10)	22 (9)	30 (15)
Self-induced vomiting (n = 6,613)	18 (6)	18 (6)	21 (9)
Laxatives (n = 5,583)	21 (8)	22 (8)	35 (17)
Diuretics (n = 1,878)	23 (9)	21 (8)	30 (16)
Fasting (n = 11,305)	22 (12)	20(10)	33 (16)
Excessive exercise (n = 8,825)	20 (9)	19 (8)	29 (12)

## Data Availability

The data in this study are available via application to the relevant study.

## Research Material Availability

See data availability statement.
