# Supplementary material for: Mapping the genetic landscape of the age at onset and severity of eating disorder symptoms
Source: Br J Psychiatry. Author manuscript; Available in PMC 2026 Nov 2. (PMC7619679; doi:10.1192/bjp.2026.10763)
Supplement: Supplementary file 1 [file EMS217253-supplement-Supplementary_Materials.pdf]

Supplementary Figures

Mapping the genetic landscape of the age at onset and severity of eating disorder symptoms

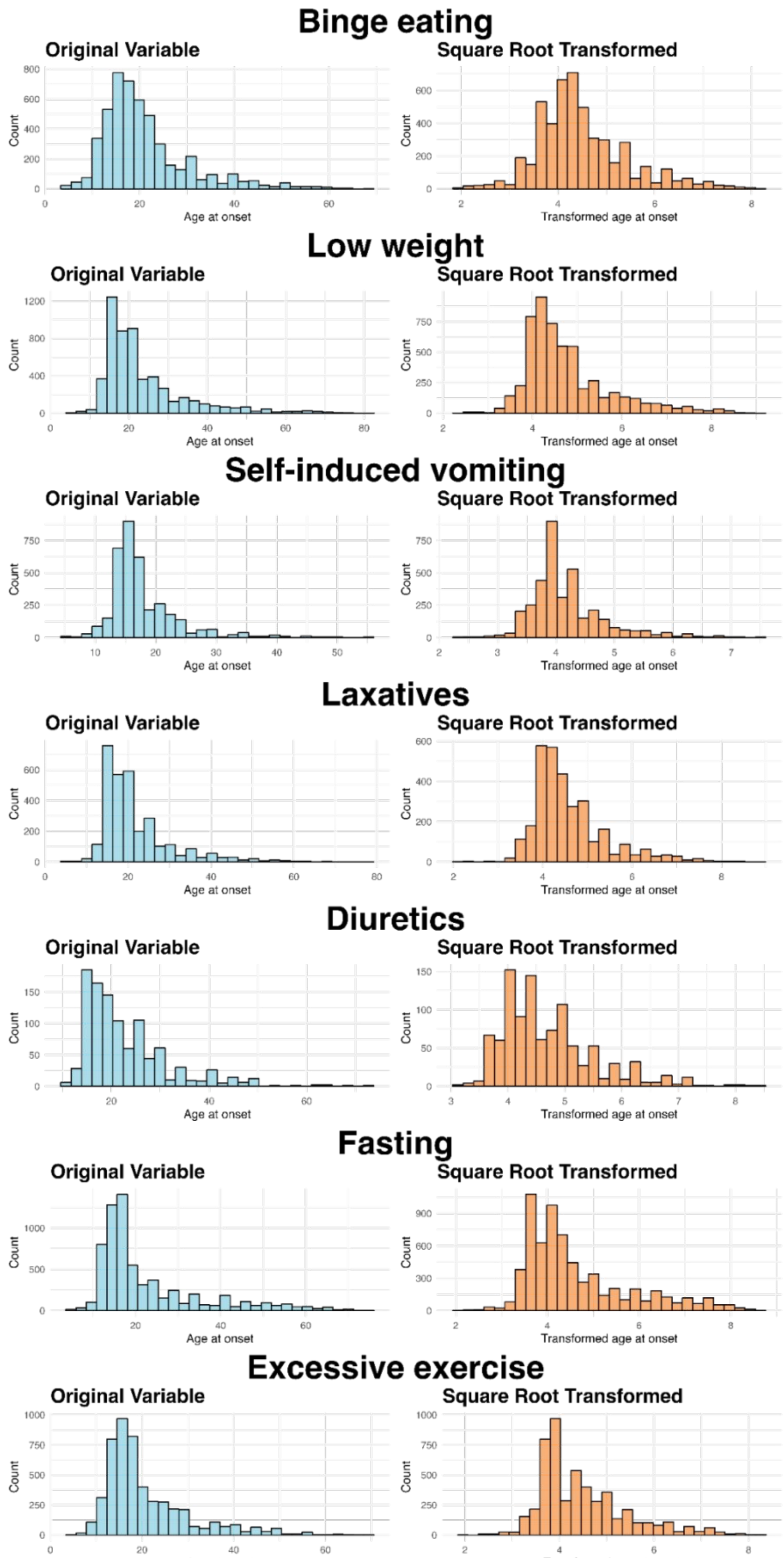

Supplementary Figure 1. Original age at onset variable for each behavioural eating disorder symptom and low weight and transformed via square root.

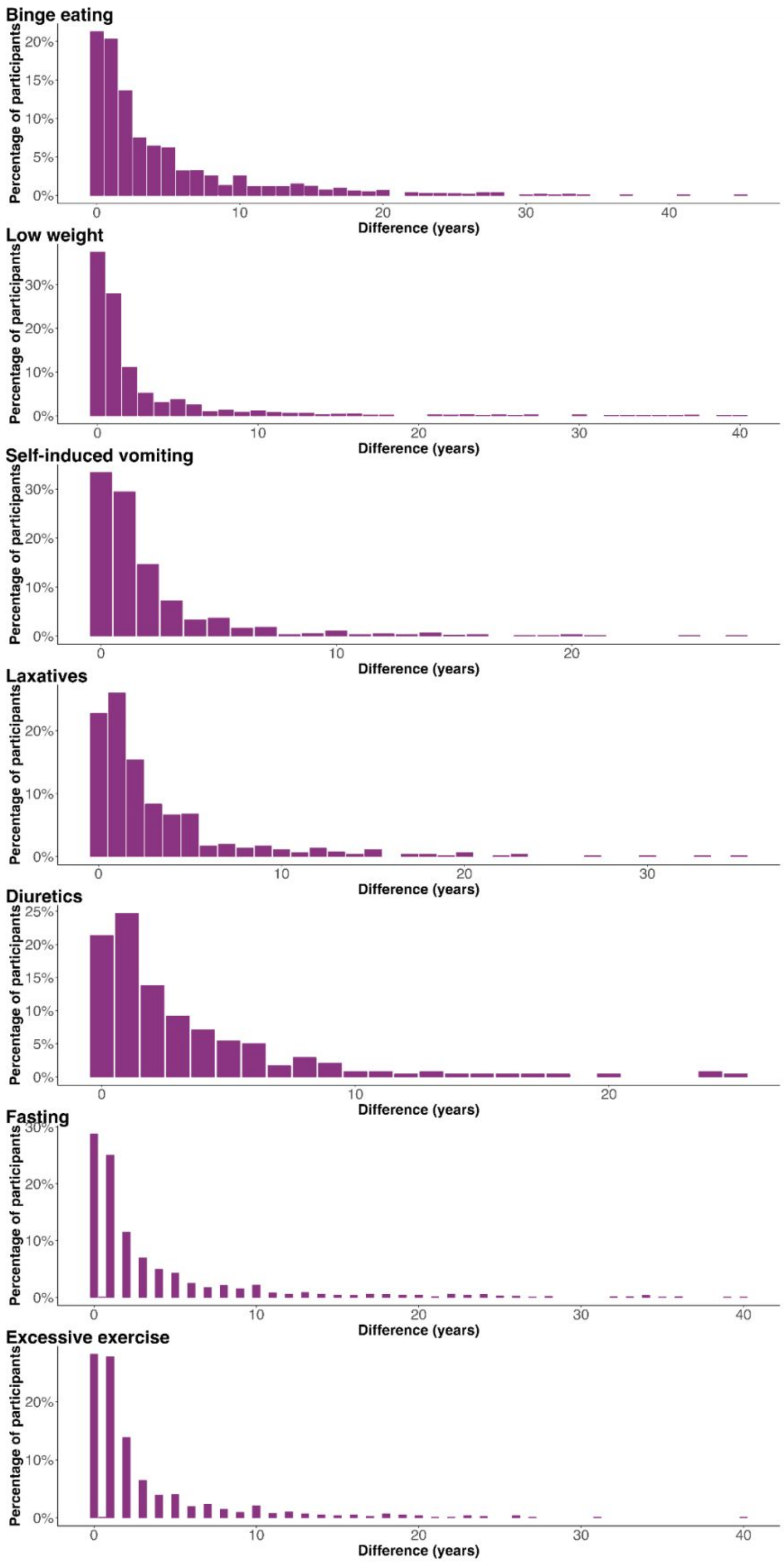

Supplementary Figure 2. Differences in reporting of age at onset in the Genetic Links to Anxiety and Depression Study and the COVID-19 Psychiatry and Neurological Genetics study.

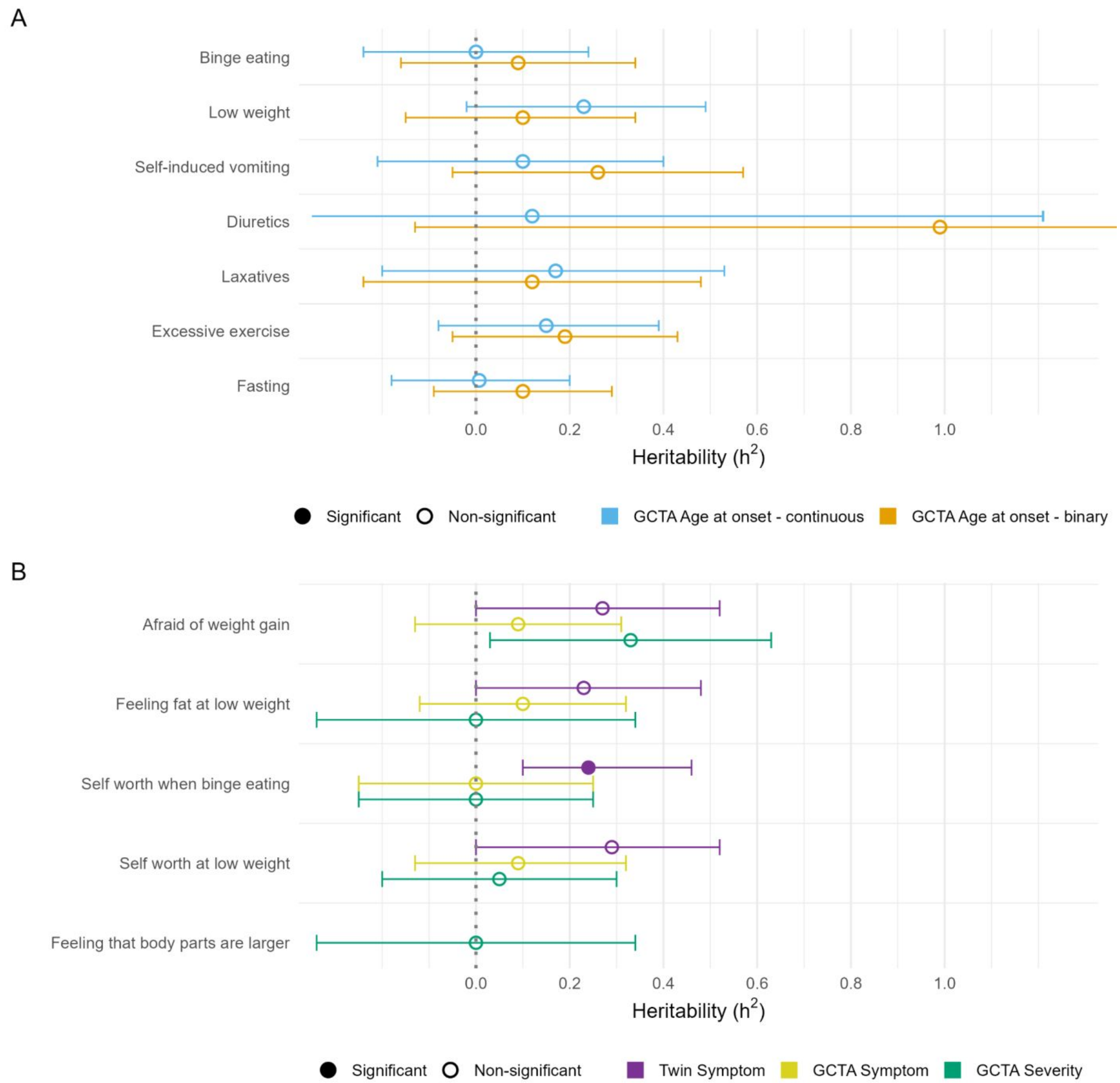

Supplementary Figure 3. (A) GCTA-GREML estimates in females of age at onset of binge eating, low weight, self-induced vomiting, diuretics, laxatives, excessive exercise, fasting and **(B)** GCTA-GREML and twin heritability estimates (where available) in females of cognitive symptoms and their severity. *Note.* We transformed age at onset variables to their square root to approach normality (Figure S1). Confidence intervals for diuretic use exceed the plot margins. Binary age at onset phenotype is coded as 1 for adult-onset (i.e., 18 years and over) and 0 for child/adolescent onset (i.e., under the age of 18). Covariates included ten principal components and genotyping batches. P-values are adjusted via False Discovery Rate within each analysis, i.e., 1) age at onset, 2) cognitive symptoms. References for twin heritability estimates are in Table S1.

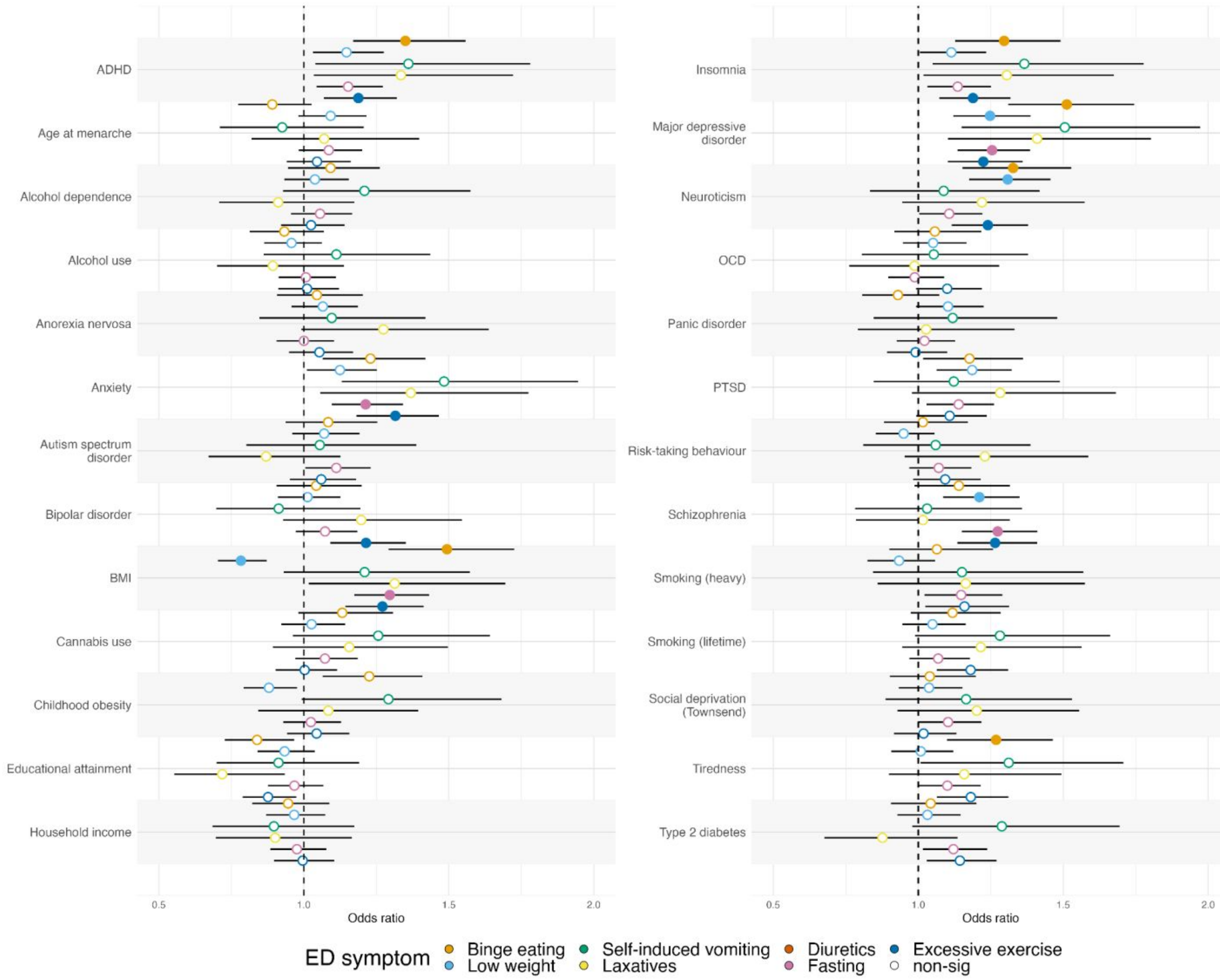

Supplementary Figure 4. Anthropometric, psychiatric, social, behavioural, somatic, and personality polygenic risk score associations with lifetime behavioural eating disorder symptoms and low weight in males.

Supplementary Table 1. Twin-based heritability of eating disorder symptoms and age at diagnosis of anorexia nervosa.

	Twin-based	Used in Figure 2
**Anorexia nervosa symptoms**		
Intentional weight loss (males)	38%[1]	
Intentional weight loss (females)	66%[1]	
Low weight (BMI < 18.5; females)	31%[2]	Yes
'Dieting' factor	42%[3]	
Afraid of weight gain during low weight	27%[2]	Yes
Feeling fat at low weight	23%[2]	Yes
Undue influence of weight on self-esteem at low weight	29%[2]	Yes
Drive for thinness (females)	51%[4]	
	44%[3]	
Drive for thinness (males)	0%[4]	
**Bulimia nervosa symptoms**		
Self-induced vomiting (females)	72%[5]	Yes
	53%[6]	
Laxatives (females)	43%[6]	Yes
Diuretics (females)	44%[6]	Yes
Diet pills (females)	51%[6]	
**Binge-eating disorder symptoms**		
Binge eating in the absence of compensatory behaviours (males &	41%[7]	
**Transdiagnostic symptoms: AN and BN**		
Fasting (females)	42%[6]	Yes
Excessive exercise (females)	35%[6]	Yes
Body dissatisfaction (females)	59%[4]	
	52%[3]	
Body dissatisfaction (males)	0%[3,4]	
Weight and shape concern (females)	25%[8]	
Importance of weight/shape to self-evaluation (females)	24%[6]	Yes
**Transdiagnostic symptoms: BN and BED**		
Binge eating (females)	34%[9]	
	46%[5]	
	34%[6]	
	49%[10]	
	70%[11]	Yes
	65%[6]	
Binge eating (males)	74%[11]*	
Binge eating (males & females)	51%[12]	
**Age at diagnosis (GCTA GREML)**		
	**SNP-based**	
Anorexia nervosa (continuous)	1%[13]	
Early-onset anorexia nervosa (i.e., binary diagnosis under the age of	21%[13]	
Typical-onset anorexia nervosa (i.e., diagnosis over the age of 13 and	15%[13]	

* The authors noted that they were only able to estimate univariate heritabilities due to small sample

Note. We have only linked symptoms to the following three eating disorders: anorexia nervosa, bulimia

References

1. Keski-Rahkonen A, Neale BM, Bulik CM, Pietiläinen KH, Rose RJ, Kaprio J, et al. Intentional weight loss in young adults: sex-specific genetic and environmental effects. Obes Res. 2005;13: 745–753.
2. Mazzeo SE, Mitchell KS, Bulik CM, Reichborn-Kjennerud T, Kendler KS, Neale MC. Assessing the heritability of anorexia nervosa symptoms using a marginal maximal likelihood approach. Psychol Med. 2009;39: 463–473.
3. Rutherford J, McGuffin P, Katz RJ, Murray RM. Genetic influences on eating attitudes in a normal female twin population. Psychol Med. 1993;23: 425–436.
4. Keski-Rahkonen A, Bulik CM, Neale BM, Rose RJ, Rissanen A, Kaprio J. Body dissatisfaction and drive for thinness in young adult twins. Int J Eat Disord. 2005;37: 188–199.
5. Curtis D. Genetic epidemiology of binging and vomiting. Br J Psychiatry. 1998;173: 439; author reply 439–40.
6. Mazzeo SE, Mitchell KS, Bulik CM, Aggen SH, Kendler KS, Neale MC. A twin study of specific bulimia nervosa symptoms. Psychol Med. 2010;40: 1203–1213.
7. Reichborn-Kjennerud T, Bulik CM, Tambs K, Harris JR. Genetic and environmental influences on binge eating in the absence of compensatory behaviors: a population-based twin study. Int J Eat Disord. 2004;36: 307–314.
8. Wade TD, Bulik CM. Shared genetic and environmental risk factors between undue influence of body shape and weight on self-evaluation and dimensions of perfectionism. Psychol Med. 2007;37: 635–644.
9. Bulik CM, Sullivan PF, Kendler KS. Heritability of binge-eating and broadly defined bulimia nervosa. Biol Psychiatry. 1998;44: 1210–1218.
10. Munn MA, Stallings MC, Rhee SH, Sobik LE, Corley RP, Rhea SA, et al. Bivariate analysis of disordered eating characteristics in adolescence and young adulthood. Int J Eat Disord. 2010;43: 751–761.
11. Root TL, Thornton LM, Lindroos AK, Stunkard AJ, Lichtenstein P, Pedersen NL, et al. Shared and unique genetic and environmental influences on binge eating and night eating: a Swedish twin study. Eat Behav. 2010;11: 92–98.
12. Reichborn-Kjennerud T, Bulik CM, Kendler KS, Røysamb E, Maes H, Tambs K, et al. Gender differences in binge-eating: a population-based twin study. Acta Psychiatr Scand. 2003;108: 196–202.
13. Watson HJ, Thornton LM, Yilmaz Z, Baker JH, Coleman JRI, Adan RAH, et al. Common Genetic Variation and Age of Onset of Anorexia Nervosa. Biol Psychiatry Glob Open Sci. 2022;2: 368–378.

Supplementary Table 2. Sub-cohorts of the National Institute for Health and

	N	Recruitment	Eligibility criteria	Recruitment
Genetic Links	7,036	Social media,	16+ years, live in	England, Wales,
Eating	5,301	Social media	16+, live in	England
Inflammatory	163	IBD clinics in	16+, have a	England, Wales,
NHS Blood	8,674	Blood donation	16+, live in	England
Research	3,799	Biomedical	16+, live in	England

Supplementary Table 3. Question items from the ED100K for each symptom and their age at

**Lifetime symptom and age at onset**	
**Binge eating**	
Symptom (binary)	1) Have you ever had regular episodes of overeating or eating binges when
Age at onset	Roughly how old were you when you began having regular episodes of
**Low weight**	
Symptom (binary)	Have you ever had a period of time when you weighed much less than
Age at onset	How old were you then?
**Excessive exercise**	
Symptom (binary)	Have you ever used any of the following to control your body shape or
Age at onset	How old were you when you first exercised to control your weight and
**Fasting**	
Symptom (binary)	Have you ever used any of the following to control your body shape or
Age at onset	How old were you when you fasted for the first time?
**Self-induced vomiting**	
Symptom	Have you ever used any of the following to control your body shape or
Age at onset	How old were you when you began self-inducing vomiting for the first time
**Laxatives**	
Symptom (binary)	Have you ever used any of the following to control your body shape or
Age at onset	How old were you when you used laxatives for the first time?
**Diuretics**	
Symptom (binary)	Have you ever used any of the following to control your body shape or
Age at onset	How old were you when you used diuretics for the first time?
**Cognitive symptom severity**	
**Fear of weight gain at low**	During the time when you were at this low weight/BMI, how afraid were
**Feeling fat at low weight**	During the time when you were at this low weight/BMI, did you still feel
**Feeling that body parts are**	During the time when you were at this low weight/BMI, did you ever
**Self-worth dependent on**	During the time when you were at this low weight/BMI, how dependent
**Self-worth dependent on**	During the time when you were binge eating, how dependent was your self-

Supplementary Table 4. Discovery genome-wide association studies used in

Trait name	PMID of discovery GWAS	Sample size
ADHD	36702997	Cases: 38,691;
Age at menarche	31768069	456,422
Alcohol dependence	30482948	Cases: 11,569;
Alcohol use (drinks per weel	30643251	941,280
Anorexia nervosa	31308545	Cases: 16,992;
Anxiety	Genome-wide association study	Cases: 94,930;
Autism spectrum disorder	30804558	Cases: 18,381;
Bipolar disorder	34002096	Cases: 41,917;
BMI	25673413	322,154
Cannabis use	33096046	Cases: 17,068;
Childhood obesity	31504550	Cases: 8613;
Educational attainment	35361970	3,037,499
Household income	27818178	112,151
Insomnia	30804565	1,331,010
Major depressive disorder	39814019	Cases: 347,453;
Neuroticism	29500382	380,506
OCD	28761083	Cases: 2,688;
Panic disorder	31712720	Cases: 2,147;
PTSD	33510476	Cases: 36,301;
Risk-taking behaviour	30643258	
Schizophrenia	35396580	Cases: 53,386;
Smoking (heavy)	28166213	46,758
Smoking (lifetime)	30643251	632,802
Social deprivation (Townsen	27818178	112,151
Tiredness	28322280	108,976
Type 2 diabetes	30054458	Cases: 62,892;

Supplementary Table 5. Sample descriptives of participants included in the entire sample (i

	Entire	Binge eating,	Diuretics, N =	Excessive	Fasting, N =	Laxatives, N =
**Age**						
Mean (SD)	45 (18)	34 (14)	39 (14)	33 (14)	35 (15)	36 (15)
Median (IQR)	46 (28, 61)	31 (23, 44)	36 (27, 49)	29 (22, 42)	31 (23, 46)	32 (23, 46)
Range	16 - 111	16 - 107	16 - 87	16 - 107	16 - 107	16 - 107
**Female**	18,357 (73.5%)	7,997 (92.0%)	1,811 (96.4%)	7,914 (89.7%)	10,013	5,352 (95.9%)
**Transgender**	267 (2.2%)	189 (2.4%)	30 (1.8%)	168 (2.2%)	226 (2.4%)	91 (1.9%)
**Highest**						
None listed	611 (2.5%)	153 (1.8%)	36 (1.9%)	153 (1.7%)	197 (1.8%)	104 (1.9%)
CSEs or	377 (1.5%)	96 (1.1%)	23 (1.2%)	69 (0.8%)	99 (0.9%)	67 (1.2%)
O-	2,869 (11.7%)	848 (9.8%)	184 (9.9%)	971 (11.1%)	1,188 (10.6%)	608 (11.0%)
AS-levels/A-	5,696 (23.2%)	2,365 (27.4%)	475 (25.6%)	2,480 (28.3%)	3,131 (27.9%)	1,523 (27.5%)
Bachelor's	8,631 (35.1%)	3,643 (42.2%)	780 (42.0%)	3,556 (40.5%)	4,421 (39.4%)	2,274 (41.0%)
NVQ/HND/HN	1895 (7.6%)	523 (6%)	142 (7.7%)	441 (5%)	646 (5.7%)	348 (6.3%)
Masters	2,464 (10.0%)	583 (6.8%)	117 (6.3%)	628 (7.2%)	879 (7.8%)	350 (6.3%)
Postgraduate	1,331 (5.4%)	283 (3.3%)	73 (3.9%)	315 (3.6%)	441 (3.9%)	195 (3.5%)
PhD	721 (2.9%)	131 (1.5%)	28 (1.5%)	160 (1.8%)	208 (1.9%)	74 (1.3%)
**Race/Ethnicit**						
Arab	15 (0.1%)	13 (0.2%)	0 (0.0%)	11 (0.1%)	12 (0.1%)	5 (0.1%)
Asian or Asian	262 (1.1%)	95 (1.1%)	16 (0.9%)	106 (1.2%)	138 (1.2%)	57 (1.0%)
Black or Black	89 (0.4%)	32 (0.4%)	9 (0.5%)	32 (0.4%)	42 (0.4%)	26 (0.5%)
Mixed	412 (1.7%)	252 (2.9%)	50 (2.7%)	246 (2.8%)	303 (2.7%)	154 (2.8%)
Other	137 (0.6%)	75 (0.9%)	18 (1.0%)	79 (0.9%)	93 (0.8%)	44 (0.8%)
White	23,478 (96.2%)	8,168 (94.6%)	1,773 (95.0%)	8,271 (94.6%)	10,616	5,256 (94.8%)

**NB: includes comparison group without psychiatric disorders*

.e., including people

Low weight, N	Vomiting, N =
37 (16)	31 (13)
31 (23, 49)	28 (21, 39)
16 - 102	16 - 77
8,381 (89.0%)	6,371 (96.3%)
157 (2.1%)	157 (2.6%)
213 (2.3%)	122 (1.9%)
111 (1.2%)	56 (0.9%)
1,105 (11.8%)	748 (11.4%)
2,584 (27.7%)	1,907 (29.0%)
3,507 (37.6%)	2,735 (41.6%)
546 (5.8%)	323 (4.9%)
706 (7.6%)	375 (5.7%)
364 (3.9%)	219 (3.3%)
203 (2.2%)	90 (1.4%)
12 (0.1%)	11 (0.2%)
130 (1.4%)	72 (1.1%)
30 (0.3%)	25 (0.4%)
253 (2.7%)	218 (3.3%)
71 (0.8%)	59 (0.9%)
8,839 (94.7%)	6,186 (94.1%)

18.3

Supplementary Table 6. Average differences between self-reported age of onset of the same partic

	Binge eating	Low weight (*n*	Self-induced	Laxative use
Mean (SD)	4.4 (5.9)	2.5 (4.8)	2 (3.2)	3.1 (4.4)
Median (IQR)	2 (4)	1 (2)	1 (2)	2 (3)
Range	0 - 45	0 - 40	0 - 27	0 - 35
n > 10 years	171 (12%)	74 (5.2%)	29 (3.1%)	54 (6.5%)
Correlation between discrepancy and age (r, *p*	0.23 (<0.001)	0.16 (<0.001)	0.14 (<0.001)	0.25 (<0.001)
Correlation between time-points (r, *p*)	0.69 (<0.001)	0.85 (<0.001)	0.81 (<0.001)	0.79 (<0.001)

Note. SD = standard deviation; IQR = interquartile range. Correlations are Spearman's rank.

ipants' answers in the GLAD Study versus

Diuretic use (*n*	Fasting (*n* =	Excessive
3.3 (4.3)	3.5 (5.7)	3 (4.7)
2 (3)	1 (4)	1 (3)
0 - 25	0 - 40	0 - 40
14 (5.9%)	153 (8.5%)	75 (7%)
0.27 (<0.001)	0.24 (<0.001)	0.28 (<0.001)
0.71 (<0.001)	0.77 (<0.001)	0.78 (<0.001)

Supplementary Table 7. Mean, median, and range of age at onset of

	Entire sample,	Female,	Male,
**Binge eating (n = 8,691)**			
Mean (SD)	20 (9)	20 (9)	25 (12)
Median (IQR)	18 (15, 24)	18 (15, 23)	22 (16, 32)
Range	4 - 78	4 - 78	5 - 70
**Low weight (n = 9,420)**			
Mean (SD)	23 (10)	22 (9)	30 (15)
Median (IQR)	20 (17, 25)	19 (16, 25)	24 (19, 38)
Range	4 - 81	4 - 78	6 - 81
**Self-induced vomiting (n =**			
Mean (SD)	18 (6)	18 (6)	21 (9)
Median (IQR)	16 (14, 19)	16 (14, 19)	18 (16, 24)
Range	5 - 65	5 - 65	5 - 55
**Laxatives (n = 5,583)**			
Mean (SD)	21 (8)	22 (8)	35 (17)
Median (IQR)	19 (16, 24)	20 (17, 25)	30 (21, 44)
Range	4 - 80	5 - 84	12 - 83
**Diuretics (n = 1,878)**			
Mean (SD)	23 (9)	21 (8)	30 (16)
Median (IQR)	20 (17, 25)	18 (16, 23)	25 (18, 40)
Range	5 - 84	4 - 70	4 - 80
**Fasting (n = 11,305)**			
Mean (SD)	22 (12)	20 (10)	33 (16)
Median (IQR)	17 (14, 25)	17 (14, 22)	30 (19, 45)
Range	4 - 75	4 - 71	5 - 75
**Excessive exercise (n = 8,825)**			
Mean (SD)	20 (9)	19 (8)	29 (12)
Median (IQR)	17 (14, 23)	17 (14, 22)	25 (18, 38)
Range	4 - 72	4 - 72	8 - 69

Supplementary Table 8. Mean, median, and range of age at onset of each

	Age at onset	Female,	Male,
**Binge eating (n = 7,778)**			
Mean (SD)	20 (8)	20 (8)	25 (12)
Median (IQR)	18 (15, 23)	18 (15, 22)	21 (16, 30)
Range	4 - 70	4 - 68	5 - 70
**Low weight (n = 8,799)**			
Mean (SD)	23 (10)	22 (9)	29 (15)
Median (IQR)	19 (16, 25)	19 (16, 24)	24 (19, 38)
Range	4 - 81	4 - 78	7 - 81
**Self-induced vomiting (n =**			
Mean (SD)	18 (6)	17 (5)	21 (9)
Median (IQR)	16 (14, 19)	16 (14, 19)	18 (16, 24)
Range	5 - 60	5 - 60	5 - 55
**Laxatives (n = 5,034)**			
Mean (SD)	21 (8)	22 (8)	35 (18)
Median (IQR)	18 (16, 23)	20 (17, 25)	29 (21, 44)
Range	4 - 80	5 - 84	12 - 83
**Diuretics (n = 1,622)**			
Mean (SD)	22 (8)	20 (7)	31 (16)
Median (IQR)	20 (17, 25)	18 (16, 23)	24 (18, 42)
Range	5 - 84	4 - 70	4 - 80
**Fasting (n = 10,343)**			
Mean (SD)	21 (12)	20 (10)	33 (16)
Median (IQR)	17 (14, 24)	16 (14, 21)	30 (19, 45)
Range	4 - 75	4 - 71	5 - 75
**Excessive exercise (n = 8,164)**			
Mean (SD)	20 (9)	19 (8)	29 (12)
Median (IQR)	17 (14, 22)	16 (14, 21)	25 (18, 38)
Range	4 - 72	4 - 72	8 - 69

Supplementary Table 9. Categorical age at onset of each phenotype split by sex: binge

	Female	Male
**Binge eating**		
Childhood: 12 years and under	864 (11%)	67 (9.7%)
Adolescence: Between 13 and 17 years	2,793 (35%)	132 (19%)
Emerging adulthood: Between 18 and 25 years	2,921 (37%)	231 (33%)
Adulthood: 26 years and over	1,419 (18%)	264 (38%)
**Low weight**		
Childhood: 12 years and under	173 (3.8%)	39 (6.6%)
Adolescence: Between 13 and 17 years	1,774 (39%)	72 (12%)
Emerging adulthood: Between 18 and 25 years	1,155 (25%)	58 (9.9%)
Adulthood: 26 years and over	1,465 (32%)	418 (71%)
**Self-induced vomiting**		
Childhood: 12 years and under	517 (13%)	15 (9.6%)
Adolescence: Between 13 and 17 years	2,124 (53%)	53 (34%)
Emerging adulthood: Between 18 and 25 years	1,000 (25%)	50 (32%)
Adulthood: 26 years and over	360 (9.0%)	39 (25%)
**Laxatives**		
Childhood: 12 years and under	111 (3.2%)	7 (4.1%)
Adolescence: Between 13 and 17 years	1,699 (49%)	37 (22%)
Emerging adulthood: Between 18 and 25 years	1,046 (30%)	37 (22%)
Adulthood: 26 years and over	647 (18%)	90 (53%)
**Diuretics**		
Childhood: 12 years and under	24 (1.9%)	1 (1.9%)
Adolescence: Between 13 and 17 years	564 (45%)	9 (17%)
Emerging adulthood: Between 18 and 25 years	403 (32%)	11 (21%)
Adulthood: 26 years and over	260 (21%)	32 (60%)
**Excessive exercise**		
Childhood: 12 years and under	766 (16%)	13 (2.2%)
Adolescence: Between 13 and 17 years	2,102 (45%)	110 (18%)
Emerging adulthood: Between 18 and 25 years	1,014 (22%)	96 (16%)
Adulthood: 26 years and over	825 (18%)	384 (64%)
**Fasting**		
Childhood: 12 years and under	956 (16%)	40 (4.2%)
Adolescence: Between 13 and 17 years	2,574 (42%)	128 (14%)
Emerging adulthood: Between 18 and 25 years	1,185 (20%)	107 (11%)
Adulthood: 26 years and over	1,349 (22%)	667 (71%)

Supplementary Table 10. Regression analysis investigating the association between gender a

			**Mean ag**
	**Binge eating**	**Low weight**	**Self-induced**
Gender			
**Man**	26 (12, [662])	30 (15, [1013])	21 (9, [229])
**Woman**	20 (9, [7625])	22 (9, [8037])	18 (6, [6047])
**Non-binary**	18 (9, [135])	21 (8, [137])	16 (5, [113])
**Self-define**	16 (6, [29])	21 (10, [37])	16 (6, [29])
**Transgender**	17 (6, [189])	18 (7, [157])	16 (4, [157])
			**Regres**
Gender as predictor (versus being a	**Binge eating**	**Low weight**	**Self-induced**
**Woman**	8.66 (8.58, 8.74), q =	7.09 (7.02,	22.06 (21.93,
Sex as predictor (versus male)			
**Female**	8.72 (8.64, 8.8), q =	7.18 (7.11,	22.05 (21.92,
			**Regress**
Gender as predictor (versus being a	**Binge eating**	**Low weight**	**Self-induced**
**Non-binary**	-7.22 (-8.86, -5.58), q	-9.36 (-11.17, -	-5.33 (-6.66, -
**Transgender**	-9.03 (-10.46, -7.6), q	-11.38 (-13.08,	-5.72 (-6.92, -
**Woman**	-5.39 (-6.09, -4.68), q	-7.54 (-8.2, -	-3.6 (-4.38, -
Sex as predictor (versus being male)			
**Female**	-5.31 (-6, -4.62), q =	-7.45 (-8.11, -	-3.58 (-4.34, -

*Regressions in which the outcome was presence or ansence of a symptom required controls, i.e., pe

and sex with behavioural symptoms and low weight and their

ge at onset (SD [n])			
Laxatives	Fasting	Excessive	Diuretics
31 (16, [216])	33 (16, [1258])	29 (12, [892])	36 (17, [59])
21 (8, [5153])	21 (11, [9538])	19 (8, [7536])	22 (8, [1741])
18 (4, [79])	17 (8, [175])	17 (6, [146])	19 (4, [32])
23 (14, [16])	18 (10, [42])	17 (9, [31])	24 (11, [<10])
18 (6, [91])	16 (7, [226])	16 (5, [168])	19 (5, [30])
**ssions: Symptom**			
**Laxatives**	**Fasting**	**Excessive**	**Diuretics**
19.27 (19.14,	6.71 (6.64, 6.78),	6.49 (6.42,	23.16 (22.91,
18.84 (18.71,	6.84 (6.77, 6.91),	6.7 (6.63, 6.78),	21.56 (21.33,
**sions: Age at onset**			
**Laxatives**	**Fasting**	**Excessive**	**Diuretics**
-12.33 (-14.44,	-15.37 (-17.14, -	-12.25 (-13.74, -	-17.84 (-21.37,
-12.94 (-14.94,	-16.36 (-17.95, -	-13.48 (-14.88, -	-17.74 (-21.35,
-9.87 (-10.98, -	-12.16 (-12.82, -	-9.69 (-10.28, -	-14.07 (-16.2, -
-9.59 (-10.67, -	-12.22 (-12.87, -	-9.66 (-10.25, -	-12.46 (-14.47,

eople without the symptom. The sample size of genders other

r

Supplementary Table 11. GCTA-GREML heritability estimates in females of

	GCTA-GREML h2 (SE;	Twin h2 (females)
**Behavioural symptoms and low weight**		
**Binge eating**		
Symptom: Liability-scale with 15%	0.43 (0.07; 0.29, 0.57),	65% (Mazzeo et al.,
Age at onset: Continuous* (n = 4,327)	0.000001 (0.13; -0.24,	NA
Age at onset: Under vs. over 18 years** (n =	0.09 (0.13; -0.16, 0.34),	NA
**Low weight**		
Symptom: Liability-scale with 17%	0.38 (0.08; 0.22, 0.53),	31% (Mazzeo et al.,
Age at onset: Continuous* (n = 4,248)	0.23 (0.13; -0.02, 0.49),	NA
Age at onset: Under vs. over 18 years** (n =	0.10 (0.12; -0.15, 0.35),	NA
**Self-induced vomiting**		
Symptom: Liability-scale with 8% prevalence	0.53 (0.07; 0.40, 0.67),	72% (Curtis, 1998)
Age at onset: Continuous* (n = 3,414)	0.10 (0.16; -0.21, 0.40),	NA
Age at onset: Under vs. over 18 years** (n =	0.26 (0.16; -0.05, 0.57),	NA
**Diuretics**		
Symptom: Liability-scale with 3% prevalence	0.78 (0.10; 0.57, 0.99),	44% (Mazzeo et al.,
Age at onset: Continuous* (n = 979)	0.12 (0.56; -0.96, 1.21),	NA
Age at onset: Under vs. over 18 years** (n =	0.99 (0.57; -0.11, 2.11),	NA
**Laxatives**		
Symptom: Liability-scale with 10%	0.43 (0.08; 0.28, 0.59),	43%
Age at onset: Continuous* (n = 2,964)	0.17 (0.19; -0.20, 0.53),	NA
Age at onset: Under vs. over 18 years** (n =	0.12 (0.18; -0.24, 0.48),	NA
**Excessive exercise**		
Symptom: Liability-scale with 15%	0.46 (0.07; 0.31, 0.60),	35% (Mazzeo et al.,
Age at onset: Continuous* (n = 4,406)	0.15 (0.12; -0.08, 0.39),	NA
Age at onset: Under vs. over 18 years** (n =	0.19 (0.12; -0.05, 0.43),	NA
**Fasting**		
Symptom: Liability-scale with 21%	0.31 (0.08; 0.16, 0.47),	42% (Mazzeo et al.,
Age at onset: Continuous* (n = 5,668)	0.007 (0.10; -0.18,	NA
Age at onset: Under vs. over 18 years** (n =	0.10 (0.09; -0.09, 0.29),	NA
**Cognitive symptoms**		
**Afraid of gaining weight (at low weight)**		
Symptom (n = 4,849; 3,592 cases)	0.09 (0.11; -0.13, 0.31),	27% (Mazzeo et al.,
Severity: 4-point scale (n = 3,653)	0.33 (0.15; 0.03, 0.63),	
**Feeling fat (at low weight)**		
Symptom (n = 4,843; 3,162 cases)	0.10 (0.11; -0.12, 0.32),	23% (Mazzeo et al.,
Severity: 4-point scale (n = 3,222)	0 (0.17; -0.34, 0.34), p	
**Self-worth dependent on body weight/shape (whilst binge eating)**		
Symptom (n = 4,439; 4,273 cases)	0 (0.13; -0.25, 0.25),	24% (Mazzeo et al.,
Severity: 6-point scale (n = 4,273)	0 (0.13; -0.25, 0.25),	
**Self-worth dependent on body weight/shape (at low weight)**		
Symptom (n = 4,841; 4,000 cases)	0.09 (0.11; -0.13, 0.32),	29% (Mazzeo et al.,
Severity: 6-point scale (n = 4,102)	0.05 (0.13; -0.20, 0.30),	
**Feeling that body parts are larger than they are (at low weight)**		
Severity: 3-point scale (n = 3,167)	0.000001 (0.17; -0.34,	NA

*We transformed continuous variables to their square root to approach normality

Supplementary Table 12. Liability-scale GCTA-GREML heritability estimates

	GCTA-GREML h2 (SE; 95% CI), p
**Binge eating (n = 9,089; 5,173 cases)**	
12%	0.40 (0.07; 0.27, 0.53), 2.91x10-9
18%	0.45 (0.08; 0.30, 0.60), 2.92x10-9
**Low weight (n = 8,500; 4,849 cases)**	
14%	0.36 (0.08; 0.21, 0.50), 2.49x10-6
20%	0.40 (0.08; 0.23, 0.56), 2.49x10-6
**Self-induced vomiting (n = 7,765; 3,490 cases)**	
6%	0.49 (0.06; 0.37, 0.61), 3.33x10-15
10%	0.57 (0.07; 0.43, 0.72), 3.33x10-15
**Diuretics (n = 5,590; 1,092 cases)**	
2%	0.70 (0.10; 0.48, 0.85), 9.47x10-13
4%	0.85 (0.12; 0.62, 1.1), 9.47x10-13
**Laxatives (n = 7,259; 3,050 cases)**	
8%	0.40 (0.07; 0.26, 0.54), 2.47x10-8
12%	0.46 (0.08; 0.29, 0.62), 2.47x10-8
**Excessive exercise (n = 8,873; 4,901 cases)**	
12%	0.43 (0.07; 0.29, 0.56), 5.55x10-10
18%	0.48 (0.08; 0.33, 0.64), 5.55x10-10
**Fasting (n = 9,555; 5,870 cases)**	
17%	0.29 (0.07; 0.14, 0.44), 8.09x10-5
25%	0.33 (0.08; 0.16, 0.49), 8.09x10-5

Note. Covariates included ten principal components and genotyping batches. We estimated

Supplementary Table 13. GCTA-GREML

	**GCTA-GREML h2 (SE), *p* [FDR-**
Binge eating	0.000001 (0.13),
Low weight	0.12 (0.13),
Self-inducted	0.08 (0.16),
Diuretics (*n*=979)	0.75 (0.55),
Laxatives (*n*=2,964)	0.14 (0.19),
Exercise (*n*=4,406)	0.16 (0.12),
Fasting (*n*=5,668)	0.000002 (0.10),

Supplementary Table 14. Associations of anthropometric, psychiatric, social, behavioural, som

PRS predictor	e eating (case *n* = 5,138; control *n* = 3	veight (case *n* = 4,752; control *n* = 3
**ADHD**	1.2 (1.1, 1.3), q = 0, (Sig)	1.12 (1.05, 1.21), q = 0.003, (Sig)
**Age at menarche**	0.84 (0.78, 0.91), q = 0, (Sig)	1.07 (1, 1.15), q = 0.074, (Non-sig)
**Alcohol dependence**	0.95 (0.87, 1.03), q = 0.238, (Non-sig)	1.03 (0.96, 1.11), q = 0.46, (Non-sig)
**Alcohol use**	0.95 (0.88, 1.03), q = 0.252, (Non-sig)	0.93 (0.87, 1), q = 0.084, (Non-sig)
**Anorexia nervosa**	0.99 (0.92, 1.08), q = 0.929, (Non-sig)	1.09 (1.02, 1.17), q = 0.028, (Non-sig)
**Anxiety**	1.18 (1.09, 1.28), q = 0, (Sig)	1.16 (1.09, 1.25), q = 0, (Sig)
**Autism spectrum disorder**	1.19 (1.1, 1.3), q = 0, (Sig)	1.09 (1.01, 1.17), q = 0.036, (Non-sig)
**BMI**	1.41 (1.3, 1.53), q = 0, (Sig)	0.76 (0.71, 0.82), q = 0, (Sig)
**Bipolar disorder**	1.11 (1.03, 1.21), q = 0.018, (Non-sig)	1.13 (1.05, 1.21), q = 0.002, (Sig)
**Cannabis use**	1.05 (0.97, 1.14), q = 0.279, (Non-sig)	1.05 (0.98, 1.13), q = 0.209, (Non-sig)
**Childhood obesity**	1.14 (1.05, 1.24), q = 0.003, (Sig)	0.85 (0.8, 0.91), q = 0, (Sig)
**Educational attainment**	0.9 (0.83, 0.98), q = 0.027, (Non-sig)	0.99 (0.92, 1.06), q = 0.795, (Non-sig)
**Household income**	0.89 (0.82, 0.96), q = 0.007, (Sig)	1.04 (0.97, 1.11), q = 0.339, (Non-sig)
**Insomnia**	1.14 (1.05, 1.24), q = 0.003, (Sig)	1.01 (0.94, 1.08), q = 0.889, (Non-sig)
**Major depressive disorder**	1.38 (1.27, 1.5), q = 0, (Sig)	1.24 (1.15, 1.33), q = 0, (Sig)
**Neuroticism**	1.16 (1.07, 1.26), q = 0.001, (Sig)	1.18 (1.1, 1.27), q = 0, (Sig)
**OCD**	1 (0.93, 1.09), q = 0.944, (Non-sig)	0.96 (0.89, 1.03), q = 0.279, (Non-sig)
**PTSD**	1.17 (1.08, 1.27), q = 0.001, (Sig)	1.15 (1.07, 1.24), q = 0, (Sig)
**Panic disorder**	0.99 (0.91, 1.07), q = 0.816, (Non-sig)	1.04 (0.97, 1.12), q = 0.289, (Non-sig)
**Risk-taking behaviour**	1.15 (1.06, 1.24), q = 0.002, (Sig)	1.02 (0.96, 1.1), q = 0.583, (Non-sig)
**Schizophrenia**	1.22 (1.12, 1.32), q = 0, (Sig)	1.15 (1.07, 1.23), q = 0.001, (Sig)
**Smoking (heavy)**	1.27 (1.16, 1.4), q = 0, (Sig)	0.94 (0.86, 1.02), q = 0.207, (Non-sig)
**Smoking (lifetime)**	1.17 (1.08, 1.27), q = 0, (Sig)	1.01 (0.94, 1.09), q = 0.809, (Non-sig)
**Social deprivation (Townsend**	1.11 (1.03, 1.2), q = 0.016, (Non-sig)	1 (0.93, 1.07), q = 0.994, (Non-sig)
**Tiredness**	1.18 (1.09, 1.28), q = 0, (Sig)	1.12 (1.04, 1.2), q = 0.004, (Sig)
**Type 2 diabetes**	1.12 (1.03, 1.21), q = 0.014, (Non-sig)	0.97 (0.9, 1.04), q = 0.49, (Non-sig)

Note. q values are rounded up to 3 decimal points.

ıatic, personality polygenic risk score associations with lifetime behavioural eating disorder symptoms

**Odds ratio (95% Confidence intervals), False Discovery Rate *q*-value**		
**ced vomiting (case *n* = 3,487; control *n***	**tives (case *n* = 3,008; control *n* = 3,**	**etics (case *n* = 1,073; control *n* = 4,**
1.24 (1.13, 1.37), q = 0, (Sig)	1.28 (1.17, 1.41), q = 0, (Sig)	1.36 (1.19, 1.55), q = 0, (Sig)
0.99 (0.9, 1.08), q = 0.828, (Non-sig)	0.9 (0.82, 0.98), q = 0.027, (Non-sig)	0.88 (0.78, 0.99), q = 0.062, (Non-sig)
1 (0.91, 1.1), q = 0.953, (Non-sig)	0.94 (0.86, 1.03), q = 0.279, (Non-sig)	0.96 (0.84, 1.09), q = 0.61, (Non-sig)
1.05 (0.96, 1.15), q = 0.344, (Non-sig)	1.03 (0.94, 1.12), q = 0.617, (Non-sig)	0.98 (0.86, 1.11), q = 0.8, (Non-sig)
1.07 (0.98, 1.18), q = 0.194, (Non-sig)	1.05 (0.96, 1.15), q = 0.321, (Non-sig)	0.91 (0.79, 1.04), q = 0.207, (Non-sig)
1.16 (1.06, 1.28), q = 0.004, (Sig)	1.13 (1.03, 1.23), q = 0.018, (Non-sig)	1.11 (0.97, 1.26), q = 0.183, (Non-sig)
1.17 (1.07, 1.29), q = 0.003, (Sig)	1.16 (1.06, 1.27), q = 0.003, (Sig)	1.1 (0.96, 1.25), q = 0.234, (Non-sig)
1.17 (1.06, 1.29), q = 0.003, (Sig)	1.19 (1.08, 1.3), q = 0.001, (Sig)	1.28 (1.12, 1.46), q = 0.001, (Sig)
1.27 (1.16, 1.4), q = 0, (Sig)	1.21 (1.11, 1.33), q = 0, (Sig)	1.14 (1, 1.3), q = 0.074, (Non-sig)
1.06 (0.96, 1.16), q = 0.3, (Non-sig)	1.1 (1.01, 1.2), q = 0.055, (Non-sig)	1.1 (0.97, 1.25), q = 0.208, (Non-sig)
1 (0.92, 1.1), q = 0.953, (Non-sig)	1.06 (0.97, 1.16), q = 0.255, (Non-sig)	0.99 (0.87, 1.13), q = 0.929, (Non-sig)
0.94 (0.86, 1.04), q = 0.283, (Non-sig)	0.82 (0.75, 0.9), q = 0, (Sig)	0.85 (0.74, 0.97), q = 0.027, (Non-sig)
0.95 (0.87, 1.04), q = 0.335, (Non-sig)	0.84 (0.77, 0.92), q = 0, (Sig)	0.86 (0.76, 0.97), q = 0.03, (Non-sig)
1.07 (0.98, 1.18), q = 0.199, (Non-sig)	1.2 (1.1, 1.31), q = 0, (Sig)	1.25 (1.1, 1.42), q = 0.002, (Sig)
1.39 (1.27, 1.53), q = 0, (Sig)	1.4 (1.28, 1.54), q = 0, (Sig)	1.29 (1.13, 1.48), q = 0.001, (Sig)
1.08 (0.98, 1.18), q = 0.189, (Non-sig)	1.25 (1.15, 1.37), q = 0, (Sig)	1.13 (0.99, 1.29), q = 0.12, (Non-sig)
0.95 (0.87, 1.04), q = 0.34, (Non-sig)	0.99 (0.9, 1.08), q = 0.828, (Non-sig)	0.93 (0.82, 1.06), q = 0.335, (Non-sig)
1.15 (1.05, 1.27), q = 0.008, (Sig)	1.24 (1.13, 1.35), q = 0, (Sig)	1.31 (1.15, 1.5), q = 0, (Sig)
1 (0.91, 1.1), q = 0.948, (Non-sig)	0.99 (0.91, 1.09), q = 0.929, (Non-sig)	1.02 (0.89, 1.16), q = 0.85, (Non-sig)
1.16 (1.06, 1.27), q = 0.004, (Sig)	1.15 (1.06, 1.26), q = 0.003, (Sig)	1.19 (1.05, 1.35), q = 0.012, (Non-sig)
1.25 (1.13, 1.38), q = 0, (Sig)	1.24 (1.13, 1.36), q = 0, (Sig)	1.09 (0.95, 1.24), q = 0.303, (Non-sig)
1.2 (1.07, 1.34), q = 0.003, (Sig)	1.24 (1.11, 1.38), q = 0, (Sig)	1.19 (1.02, 1.39), q = 0.048, (Non-sig)
1.15 (1.05, 1.26), q = 0.008, (Sig)	1.23 (1.13, 1.35), q = 0, (Sig)	1.17 (1.03, 1.33), q = 0.03, (Non-sig)
1.12 (1.02, 1.23), q = 0.025, (Non-sig)	1.07 (0.99, 1.17), q = 0.155, (Non-sig)	1.12 (0.98, 1.27), q = 0.128, (Non-sig)
1.2 (1.09, 1.31), q = 0, (Sig)	1.2 (1.1, 1.31), q = 0, (Sig)	1.36 (1.19, 1.54), q = 0, (Sig)
1.01 (0.92, 1.11), q = 0.868, (Non-sig)	1.06 (0.97, 1.16), q = 0.24, (Non-sig)	1.21 (1.07, 1.38), q = 0.007, (Sig)

; and low weight in females.

**;sting (case *n* = 5,801; control *n* = 3,27:**	**exercise (case *n* = 4,878; control *n* = 3,512)**
1.14 (1.06, 1.23), q = 0.001, (Sig)	1.19 (1.1, 1.29), q = 0, (Sig)
0.88 (0.82, 0.94), q = 0, (Sig)	0.87 (0.81, 0.94), q = 0.001, (Sig)
0.95 (0.89, 1.02), q = 0.212, (Non-sig)	0.97 (0.89, 1.05), q = 0.487, (Non-sig)
0.97 (0.91, 1.04), q = 0.527, (Non-sig)	0.98 (0.91, 1.06), q = 0.704, (Non-sig)
1.02 (0.96, 1.1), q = 0.595, (Non-sig)	1.16 (1.07, 1.25), q = 0.001, (Sig)
1.12 (1.04, 1.2), q = 0.005, (Sig)	1.15 (1.06, 1.24), q = 0.002, (Sig)
1.11 (1.03, 1.19), q = 0.008, (Sig)	1.16 (1.08, 1.26), q = 0, (Sig)
1.2 (1.11, 1.29), q = 0, (Sig)	1.19 (1.1, 1.29), q = 0, (Sig)
1.11 (1.03, 1.19), q = 0.008, (Sig)	1.18 (1.09, 1.27), q = 0, (Sig)
1.06 (0.98, 1.13), q = 0.189, (Non-sig)	1.09 (1.01, 1.18), q = 0.05, (Non-sig)
1.08 (1, 1.15), q = 0.059, (Non-sig)	1.1 (1.02, 1.19), q = 0.022, (Non-sig)
0.94 (0.88, 1.01), q = 0.151, (Non-sig)	0.9 (0.84, 0.98), q = 0.022, (Non-sig)
0.97 (0.91, 1.04), q = 0.544, (Non-sig)	0.91 (0.85, 0.99), q = 0.036, (Non-sig)
1.1 (1.03, 1.18), q = 0.012, (Non-sig)	1 (0.93, 1.08), q = 0.964, (Non-sig)
1.23 (1.15, 1.32), q = 0, (Sig)	1.31 (1.21, 1.42), q = 0, (Sig)
1.07 (1, 1.15), q = 0.076, (Non-sig)	1.17 (1.08, 1.26), q = 0, (Sig)
1.01 (0.94, 1.08), q = 0.816, (Non-sig)	1.01 (0.94, 1.09), q = 0.85, (Non-sig)
1.17 (1.08, 1.25), q = 0, (Sig)	1.15 (1.06, 1.24), q = 0.002, (Sig)
0.98 (0.92, 1.06), q = 0.744, (Non-sig)	1 (0.92, 1.08), q = 0.944, (Non-sig)
1.11 (1.03, 1.18), q = 0.008, (Sig)	1.11 (1.03, 1.2), q = 0.011, (Non-sig)
1.13 (1.05, 1.21), q = 0.002, (Sig)	1.15 (1.06, 1.24), q = 0.002, (Sig)
1.14 (1.05, 1.23), q = 0.006, (Sig)	1.25 (1.14, 1.37), q = 0, (Sig)
1.21 (1.13, 1.3), q = 0, (Sig)	1.18 (1.09, 1.27), q = 0, (Sig)
1.13 (1.06, 1.21), q = 0.001, (Sig)	1.13 (1.05, 1.22), q = 0.004, (Sig)
1.15 (1.07, 1.23), q = 0, (Sig)	1.14 (1.05, 1.23), q = 0.003, (Sig)
1.08 (1.01, 1.16), q = 0.042, (Non-sig)	1.07 (0.99, 1.16), q = 0.111, (Non-sig)

Supplementary Table 15. Associations of anthropometric, psychiatric, social, behavioural, so

	Odds ratio (95% Confidence in	
**PRS predictor**	**e eating (case *n* = 511; control *n* = 2**	**weight (case *n* = 613; control *n* = 2,**
**ADHD**	1.35 (1.17, 1.56), q = 0.001, (Sig)	1.15 (1.03, 1.28), q = 0.053, (Non-sig)
**Age at menarche**	0.89 (0.77, 1.03), q = 0.242, (Non-sig)	1.09 (0.98, 1.22), q = 0.239, (Non-sig)
**Alcohol dependence**	1.09 (0.94, 1.26), q = 0.422, (Non-sig)	1.04 (0.93, 1.16), q = 0.644, (Non-sig)
**Alcohol use**	0.93 (0.81, 1.07), q = 0.505, (Non-sig)	0.96 (0.86, 1.06), q = 0.594, (Non-sig)
**Anorexia nervosa**	1.05 (0.91, 1.2), q = 0.664, (Non-sig)	1.07 (0.96, 1.19), q = 0.441, (Non-sig)
**Anxiety**	1.23 (1.07, 1.42), q = 0.025, (Non-sig)	1.12 (1.01, 1.25), q = 0.103, (Non-sig)
**Autism spectrum disorder**	1.08 (0.94, 1.25), q = 0.473, (Non-sig)	1.07 (0.96, 1.19), q = 0.401, (Non-sig)
**BMI**	1.49 (1.29, 1.73), q = 0, (Sig)	0.78 (0.7, 0.87), q = 0, (Sig)
**Bipolar disorder**	1.04 (0.91, 1.2), q = 0.675, (Non-sig)	1.01 (0.91, 1.13), q = 0.88, (Non-sig)
**Cannabis use**	1.13 (0.98, 1.31), q = 0.212, (Non-sig)	1.03 (0.92, 1.14), q = 0.723, (Non-sig)
**Childhood obesity**	1.23 (1.07, 1.41), q = 0.025, (Non-sig)	0.88 (0.79, 0.98), q = 0.063, (Non-sig)
**Educational attainment**	0.84 (0.73, 0.97), q = 0.063, (Non-sig)	0.93 (0.84, 1.04), q = 0.375, (Non-sig)
**Household income**	0.95 (0.82, 1.09), q = 0.596, (Non-sig)	0.97 (0.87, 1.07), q = 0.661, (Non-sig)
**Insomnia**	1.3 (1.13, 1.49), q = 0.003, (Sig)	1.11 (1.01, 1.23), q = 0.119, (Non-sig)
**Major depressive disorder**	1.51 (1.31, 1.74), q = 0, (Sig)	1.25 (1.12, 1.39), q = 0.001, (Sig)
**Neuroticism**	1.33 (1.15, 1.53), q = 0.001, (Sig)	1.31 (1.18, 1.46), q = 0, (Sig)
**OCD**	1.06 (0.92, 1.22), q = 0.6, (Non-sig)	1.05 (0.95, 1.17), q = 0.531, (Non-sig)
**PTSD**	1.18 (1.02, 1.36), q = 0.096, (Non-sig)	1.19 (1.06, 1.32), q = 0.014, (Non-sig)
**Panic disorder**	0.93 (0.81, 1.07), q = 0.505, (Non-sig)	1.1 (0.99, 1.23), q = 0.175, (Non-sig)
**Risk-taking behaviour**	1.02 (0.88, 1.17), q = 0.883, (Non-sig)	0.95 (0.85, 1.06), q = 0.524, (Non-sig)
**Schizophrenia**	1.14 (0.99, 1.32), q = 0.179, (Non-sig)	1.21 (1.09, 1.35), q = 0.005, (Sig)
**Smoking (heavy)**	1.06 (0.9, 1.26), q = 0.629, (Non-sig)	0.93 (0.82, 1.06), q = 0.474, (Non-sig)
**Smoking (lifetime)**	1.12 (0.97, 1.28), q = 0.244, (Non-sig)	1.05 (0.95, 1.16), q = 0.552, (Non-sig)
**Social deprivation (Townsend**	1.04 (0.9, 1.2), q = 0.701, (Non-sig)	1.04 (0.93, 1.15), q = 0.647, (Non-sig)
**Tiredness**	1.27 (1.1, 1.46), q = 0.009, (Sig)	1.01 (0.91, 1.12), q = 0.912, (Non-sig)
**Type 2 diabetes**	1.04 (0.91, 1.2), q = 0.675, (Non-sig)	1.03 (0.93, 1.15), q = 0.675, (Non-sig)

Note. q values are rounded up to 3 decimal points.

matic, personality polygenic risk score associations with lifetime eating disorder symptoms in male

**ntervals), False Discovery Rate *q*-value**		
**:ed vomiting (case *n* = 134; control**	**atives (case *n* = 132; control *n* = 3,1**	**sting (case *n* = 784; control *n* = 2,82**
1.36 (1.04, 1.78), q = 0.086, (Non-sig)	1.33 (1.03, 1.72), q = 0.089, (Non-sig)	1.15 (1.04, 1.27), q = 0.025, (Non-sig)
0.93 (0.71, 1.21), q = 0.675, (Non-sig)	1.07 (0.82, 1.4), q = 0.723, (Non-sig)	1.09 (0.98, 1.2), q = 0.239, (Non-sig)
1.21 (0.93, 1.57), q = 0.321, (Non-sig)	0.91 (0.71, 1.17), q = 0.633, (Non-sig)	1.06 (0.96, 1.17), q = 0.476, (Non-sig)
1.11 (0.86, 1.44), q = 0.594, (Non-sig)	0.89 (0.7, 1.14), q = 0.548, (Non-sig)	1.01 (0.91, 1.11), q = 0.918, (Non-sig)
1.1 (0.85, 1.42), q = 0.642, (Non-sig)	1.27 (0.99, 1.64), q = 0.157, (Non-sig)	1 (0.91, 1.1), q = 0.995, (Non-sig)
1.48 (1.13, 1.95), q = 0.025, (Non-sig)	1.37 (1.06, 1.77), q = 0.069, (Non-sig)	1.21 (1.1, 1.34), q = 0.002, (Sig)
1.06 (0.8, 1.39), q = 0.779, (Non-sig)	0.87 (0.67, 1.13), q = 0.477, (Non-sig)	1.11 (1.01, 1.23), q = 0.119, (Non-sig)
1.21 (0.93, 1.57), q = 0.318, (Non-sig)	1.31 (1.02, 1.7), q = 0.113, (Non-sig)	1.3 (1.17, 1.43), q = 0, (Sig)
0.91 (0.7, 1.2), q = 0.648, (Non-sig)	1.2 (0.93, 1.55), q = 0.321, (Non-sig)	1.07 (0.97, 1.18), q = 0.321, (Non-sig)
1.26 (0.96, 1.64), q = 0.22, (Non-sig)	1.16 (0.89, 1.5), q = 0.472, (Non-sig)	1.07 (0.97, 1.19), q = 0.325, (Non-sig)
1.29 (0.99, 1.68), q = 0.157, (Non-sig)	1.08 (0.84, 1.4), q = 0.661, (Non-sig)	1.02 (0.93, 1.13), q = 0.723, (Non-sig)
0.91 (0.7, 1.19), q = 0.647, (Non-sig)	0.72 (0.55, 0.93), q = 0.059, (Non-sig)	0.97 (0.88, 1.07), q = 0.648, (Non-sig)
0.9 (0.68, 1.17), q = 0.596, (Non-sig)	0.9 (0.7, 1.17), q = 0.596, (Non-sig)	0.98 (0.88, 1.08), q = 0.723, (Non-sig)
1.37 (1.05, 1.78), q = 0.075, (Non-sig)	1.31 (1.02, 1.67), q = 0.113, (Non-sig)	1.14 (1.03, 1.25), q = 0.047, (Non-sig)
1.51 (1.15, 1.97), q = 0.018, (Non-sig)	1.41 (1.1, 1.8), q = 0.031, (Non-sig)	1.25 (1.14, 1.39), q = 0, (Sig)
1.09 (0.83, 1.42), q = 0.664, (Non-sig)	1.22 (0.95, 1.57), q = 0.268, (Non-sig)	1.11 (1, 1.22), q = 0.126, (Non-sig)
1.05 (0.81, 1.38), q = 0.779, (Non-sig)	0.99 (0.76, 1.28), q = 0.938, (Non-sig)	0.99 (0.9, 1.09), q = 0.873, (Non-sig)
1.12 (0.85, 1.49), q = 0.596, (Non-sig)	1.28 (0.98, 1.68), q = 0.175, (Non-sig)	1.14 (1.03, 1.26), q = 0.056, (Non-sig)
1.12 (0.85, 1.48), q = 0.596, (Non-sig)	1.03 (0.79, 1.33), q = 0.887, (Non-sig)	1.02 (0.93, 1.13), q = 0.759, (Non-sig)
1.06 (0.81, 1.39), q = 0.759, (Non-sig)	1.23 (0.95, 1.59), q = 0.242, (Non-sig)	1.07 (0.97, 1.18), q = 0.345, (Non-sig)
1.03 (0.78, 1.36), q = 0.883, (Non-sig)	1.02 (0.79, 1.32), q = 0.927, (Non-sig)	1.27 (1.15, 1.41), q = 0, (Sig)
1.15 (0.84, 1.57), q = 0.558, (Non-sig)	1.16 (0.86, 1.57), q = 0.512, (Non-sig)	1.15 (1.02, 1.29), q = 0.075, (Non-sig)
1.28 (0.99, 1.66), q = 0.162, (Non-sig)	1.22 (0.94, 1.56), q = 0.269, (Non-sig)	1.07 (0.97, 1.18), q = 0.345, (Non-sig)
1.16 (0.89, 1.53), q = 0.473, (Non-sig)	1.2 (0.93, 1.55), q = 0.321, (Non-sig)	1.1 (1, 1.22), q = 0.155, (Non-sig)
1.31 (1.01, 1.71), q = 0.126, (Non-sig)	1.16 (0.9, 1.49), q = 0.453, (Non-sig)	1.1 (1, 1.21), q = 0.157, (Non-sig)
1.29 (0.98, 1.69), q = 0.175, (Non-sig)	0.88 (0.68, 1.14), q = 0.505, (Non-sig)	1.12 (1.02, 1.24), q = 0.083, (Non-sig)

es.

**/e exercise (case *n* = 631; control *n* = 2,870)**
1.19 (1.07, 1.32), q = 0.01, (Sig)
1.05 (0.94, 1.16), q = 0.594, (Non-sig)
1.03 (0.92, 1.14), q = 0.74, (Non-sig)
1.01 (0.91, 1.12), q = 0.883, (Non-sig)
1.05 (0.95, 1.17), q = 0.512, (Non-sig)
1.32 (1.18, 1.47), q = 0, (Sig)
1.06 (0.95, 1.18), q = 0.476, (Non-sig)
1.27 (1.14, 1.41), q = 0, (Sig)
1.21 (1.09, 1.35), q = 0.003, (Sig)
1 (0.9, 1.11), q = 0.963, (Non-sig)
1.04 (0.94, 1.16), q = 0.594, (Non-sig)
0.88 (0.79, 0.97), q = 0.06, (Non-sig)
1 (0.9, 1.11), q = 0.952, (Non-sig)
1.19 (1.07, 1.32), q = 0.008, (Sig)
1.22 (1.1, 1.36), q = 0.002, (Sig)
1.24 (1.12, 1.38), q = 0.001, (Sig)
1.1 (0.99, 1.22), q = 0.175, (Non-sig)
1.11 (0.99, 1.24), q = 0.17, (Non-sig)
0.99 (0.89, 1.1), q = 0.899, (Non-sig)
1.09 (0.98, 1.22), q = 0.235, (Non-sig)
1.26 (1.13, 1.41), q = 0, (Sig)
1.16 (1.02, 1.31), q = 0.075, (Non-sig)
1.18 (1.06, 1.31), q = 0.012, (Non-sig)
1.02 (0.92, 1.13), q = 0.811, (Non-sig)
1.18 (1.06, 1.31), q = 0.012, (Non-sig)
1.14 (1.03, 1.27), q = 0.056, (Non-sig)

Supplementary Table 16. Associations of anthropometric, psychiatric, social, behaviour

PRS predictor	Binge eating onset (*n* = 4,423)	Low weight onset (n = 4,646)
**ADHD**	0.04 (-0.22, 0.3), q = 0.868, (Non-sig)	.09 (-0.17, 0.35), q = 0.778, (Non-sig)
**Age at menarche**	0.25 (0, 0.51), q = 0.276, (Non-sig)	).29 (0.02, 0.55), q = 0.222, (Non-sig)
**Alcohol dependence**	.32 (-0.59, -0.05), q = 0.177, (Non-sig)	0.17 (-0.1, 0.44), q = 0.506, (Non-sig)
**Alcohol use**	).19 (-0.45, 0.07), q = 0.443, (Non-sig)	.09 (-0.35, 0.18), q = 0.778, (Non-sig)
**Anorexia nervosa**	).22 (-0.48, 0.04), q = 0.374, (Non-sig)	0.1 (-0.16, 0.36), q = 0.748, (Non-sig)
**Anxiety**	).11 (-0.16, 0.38), q = 0.732, (Non-sig)	).44 (0.17, 0.71), q = 0.063, (Non-sig)
**Autism spectrum disorder**	).08 (-0.34, 0.18), q = 0.806, (Non-sig)	.21 (-0.47, 0.06), q = 0.387, (Non-sig)
**BMI**	.34 (-0.59, -0.08), q = 0.144, (Non-sig)	0.22 (-0.5, 0.05), q = 0.381, (Non-sig)
**Bipolar disorder**	).01 (-0.27, 0.25), q = 0.956, (Non-sig)	.26 (-0.52, 0.01), q = 0.276, (Non-sig)
**Cannabis use**	-0.15 (-0.4, 0.11), q = 0.6, (Non-sig)	.02 (-0.24, 0.29), q = 0.944, (Non-sig)
**Childhood obesity**	-0.58 (-0.83, -0.32), q = 0.002, (Sig)	.07 (-0.19, 0.33), q = 0.814, (Non-sig)
**Educational attainment**	0.4 (-0.67, -0.14), q = 0.071, (Non-sig)	-0.54 (-0.81, -0.27), q = 0.007, (Sig)
**Household income**	).07 (-0.32, 0.19), q = 0.815, (Non-sig)	).37 (-0.63, -0.1), q = 0.126, (Non-sig)
**Insomnia**	).07 (-0.19, 0.33), q = 0.814, (Non-sig)	.04 (-0.22, 0.31), q = 0.869, (Non-sig)
**Major depressive disorder**	).15 (-0.12, 0.41), q = 0.603, (Non-sig)	.19 (-0.08, 0.47), q = 0.443, (Non-sig)
**Neuroticism**	0.29 (0.03, 0.55), q = 0.222, (Non-sig)	.25 (-0.02, 0.51), q = 0.279, (Non-sig)
**OCD**	).12 (-0.14, 0.38), q = 0.709, (Non-sig)	.02 (-0.28, 0.24), q = 0.944, (Non-sig)
**PTSD**	).22 (-0.05, 0.49), q = 0.381, (Non-sig)	.26 (-0.01, 0.54), q = 0.276, (Non-sig)
**Panic disorder**	0.32 (0.07, 0.57), q = 0.157, (Non-sig)	.08 (-0.35, 0.19), q = 0.814, (Non-sig)
**Risk-taking behaviour**	).01 (-0.25, 0.27), q = 0.965, (Non-sig)	.09 (-0.17, 0.35), q = 0.778, (Non-sig)
**Schizophrenia**	.35 (-0.61, -0.08), q = 0.144, (Non-sig)	-0.27 (-0.54, 0), q = 0.276, (Non-sig)
**Smoking (heavy)**	).02 (-0.33, 0.29), q = 0.948, (Non-sig)	).34 (0.02, 0.65), q = 0.227, (Non-sig)
**Smoking (lifetime)**	).07 (-0.33, 0.19), q = 0.814, (Non-sig)	0.24 (-0.02, 0.51), q = 0.3, (Non-sig)
**Social deprivation (Towns**	).18 (-0.08, 0.44), q = 0.451, (Non-sig)	).41 (0.15, 0.67), q = 0.063, (Non-sig)
**Tiredness**	).21 (-0.05, 0.47), q = 0.381, (Non-sig)	.23 (-0.04, 0.49), q = 0.374, (Non-sig)
**Type 2 diabetes**	).16 (-0.42, 0.09), q = 0.506, (Non-sig)	.25 (-0.51, 0.01), q = 0.276, (Non-sig)

Note. *q values are rounded up to 3 decimal points.*

ral, somatic, personality polygenic risk score associations with age at onset of eating disorder symp

**Beta (95% Confidence intervals), False Discovery Rate *q*-value**		
**elf-induced vomiting onset (n = 3,400**	**Laxatives onset (n = 2,917)**	**Diuretics onset (n = 962)**
-0.07 (-0.26, 0.13), q = 0.778, (Non-sig)	0.07 (-0.2, 0.35), q = 0.814, (Non-sig)	).08 (-0.57, 0.41), q = 0.869, (Non-sig)
0.29 (0.1, 0.47), q = 0.071, (Non-sig)	0.31 (0.03, 0.58), q = 0.222, (Non-sig)	0.07 (-0.4, 0.53), q = 0.889, (Non-sig)
0.05 (-0.15, 0.24), q = 0.832, (Non-sig)	).08 (-0.21, 0.37), q = 0.814, (Non-sig)	).17 (-0.34, 0.69), q = 0.778, (Non-sig)
-0.07 (-0.26, 0.12), q = 0.778, (Non-sig)	).01 (-0.29, 0.27), q = 0.956, (Non-sig)	-0.3 (-0.78, 0.17), q = 0.516, (Non-sig)
-0.05 (-0.24, 0.14), q = 0.814, (Non-sig)	0.38 (-0.65, -0.1), q = 0.126, (Non-sig)	-0.2 (-0.68, 0.27), q = 0.732, (Non-sig)
-0.14 (-0.34, 0.05), q = 0.436, (Non-sig)	).06 (-0.22, 0.35), q = 0.835, (Non-sig)	).27 (-0.22, 0.76), q = 0.603, (Non-sig)
-0.14 (-0.33, 0.05), q = 0.443, (Non-sig)	).15 (-0.43, 0.13), q = 0.612, (Non-sig)	-0.2 (-0.69, 0.28), q = 0.732, (Non-sig)
-0.16 (-0.35, 0.03), q = 0.381, (Non-sig)	0 (-0.28, 0.28), q = 0.996, (Non-sig)	).04 (-0.54, 0.46), q = 0.944, (Non-sig)
-0.23 (-0.42, -0.04), q = 0.177, (Non-sig)	).03 (-0.31, 0.25), q = 0.905, (Non-sig)	).12 (-0.61, 0.37), q = 0.831, (Non-sig)
-0.06 (-0.25, 0.13), q = 0.778, (Non-sig)	0.12 (-0.15, 0.4), q = 0.726, (Non-sig)	.55 (-1.04, -0.07), q = 0.219, (Non-sig)
-0.18 (-0.37, 0.01), q = 0.279, (Non-sig)	.28 (-0.55, -0.01), q = 0.227, (Non-sig)	.49 (-0.95, -0.03), q = 0.227, (Non-sig)
-0.11 (-0.31, 0.08), q = 0.582, (Non-sig)	).23 (-0.52, 0.06), q = 0.381, (Non-sig)	).17 (-0.68, 0.34), q = 0.778, (Non-sig)
-0.08 (-0.27, 0.11), q = 0.726, (Non-sig)	).14 (-0.42, 0.14), q = 0.669, (Non-sig)	).18 (-0.67, 0.31), q = 0.768, (Non-sig)
-0.04 (-0.24, 0.15), q = 0.835, (Non-sig)	0.01 (-0.27, 0.3), q = 0.956, (Non-sig)	).06 (-0.54, 0.43), q = 0.906, (Non-sig)
-0.15 (-0.35, 0.05), q = 0.394, (Non-sig)	).12 (-0.41, 0.17), q = 0.732, (Non-sig)	).14 (-0.63, 0.35), q = 0.814, (Non-sig)
0 (-0.2, 0.19), q = 0.996, (Non-sig)	).28 (-0.01, 0.57), q = 0.276, (Non-sig)	).47 (-0.02, 0.95), q = 0.276, (Non-sig)
0.04 (-0.15, 0.23), q = 0.835, (Non-sig)	).15 (-0.13, 0.42), q = 0.612, (Non-sig)	0.4 (-0.07, 0.87), q = 0.369, (Non-sig)
-0.14 (-0.33, 0.06), q = 0.455, (Non-sig)	).14 (-0.43, 0.15), q = 0.675, (Non-sig)	).31 (-0.82, 0.19), q = 0.516, (Non-sig)
0.21 (0.02, 0.4), q = 0.222, (Non-sig)	).05 (-0.23, 0.33), q = 0.859, (Non-sig)	-0.13 (-0.6, 0.34), q = 0.814, (Non-sig)
-0.18 (-0.38, 0.01), q = 0.276, (Non-sig)	).06 (-0.33, 0.22), q = 0.835, (Non-sig)	0.02 (-0.46, 0.5), q = 0.956, (Non-sig)
-0.24 (-0.44, -0.04), q = 0.175, (Non-sig)	).21 (-0.49, 0.07), q = 0.431, (Non-sig)	0.52 (-1.02, -0.02), q = 0.23, (Non-sig)
-0.06 (-0.28, 0.17), q = 0.821, (Non-sig)	).14 (-0.47, 0.19), q = 0.732, (Non-sig)	).47 (-1.06, 0.11), q = 0.381, (Non-sig)
-0.21 (-0.4, -0.01), q = 0.227, (Non-sig)	0.02 (-0.26, 0.3), q = 0.944, (Non-sig)	-0.08 (-0.56, 0.4), q = 0.868, (Non-sig)
0.02 (-0.17, 0.21), q = 0.9, (Non-sig)	).16 (-0.11, 0.44), q = 0.572, (Non-sig)	-0.1 (-0.61, 0.42), q = 0.85, (Non-sig)
0.12 (-0.07, 0.31), q = 0.516, (Non-sig)	0.33 (0.05, 0.6), q = 0.184, (Non-sig)	).17 (-0.31, 0.65), q = 0.778, (Non-sig)
-0.04 (-0.22, 0.15), q = 0.842, (Non-sig)	).08 (-0.19, 0.35), q = 0.814, (Non-sig)	).29 (-0.17, 0.76), q = 0.516, (Non-sig)

ptoms in females.

Fasting onset (n = 5,585)	xcessive exercise onset (n = 4,379)
.05 (-0.21, 0.32), q = 0.847, (Non-sig)	).11 (-0.13, 0.34), q = 0.726, (Non-sig)
).35 (0.09, 0.61), q = 0.126, (Non-sig)	0.37 (0.14, 0.6), q = 0.063, (Non-sig)
0.02 (-0.25, 0.28), q = 0.95, (Non-sig)	).03 (-0.27, 0.21), q = 0.895, (Non-sig)
.11 (-0.15, 0.37), q = 0.732, (Non-sig)	).05 (-0.19, 0.29), q = 0.835, (Non-sig)
).36 (-0.62, -0.1), q = 0.124, (Non-sig)	-0.17 (-0.4, 0.07), q = 0.451, (Non-sig)
0.1 (-0.17, 0.37), q = 0.768, (Non-sig)	).19 (-0.05, 0.43), q = 0.387, (Non-sig)
.08 (-0.34, 0.19), q = 0.814, (Non-sig)	-0.1 (-0.34, 0.13), q = 0.732, (Non-sig)
0.1 (-0.17, 0.36), q = 0.768, (Non-sig)	).23 (-0.01, 0.46), q = 0.276, (Non-sig)
31 (-0.58, -0.05), q = 0.188, (Non-sig)	).19 (-0.43, 0.05), q = 0.387, (Non-sig)
.11 (-0.16, 0.37), q = 0.732, (Non-sig)	).03 (-0.21, 0.26), q = 0.916, (Non-sig)
0.1 (-0.36, 0.16), q = 0.768, (Non-sig)	).19 (-0.42, 0.04), q = 0.381, (Non-sig)
.19 (-0.46, 0.09), q = 0.455, (Non-sig)	.27 (-0.51, -0.02), q = 0.222, (Non-sig)
.05 (-0.21, 0.31), q = 0.847, (Non-sig)	0.17 (-0.07, 0.4), q = 0.451, (Non-sig)
0.03 (-0.24, 0.3), q = 0.905, (Non-sig)	0.07 (-0.17, 0.3), q = 0.814, (Non-sig)
.06 (-0.21, 0.33), q = 0.832, (Non-sig)	0.1 (-0.14, 0.34), q = 0.732, (Non-sig)
.02 (-0.25, 0.28), q = 0.944, (Non-sig)	).06 (-0.18, 0.29), q = 0.831, (Non-sig)
.14 (-0.12, 0.41), q = 0.603, (Non-sig)	0 (-0.23, 0.24), q = 0.996, (Non-sig)
.06 (-0.22, 0.33), q = 0.835, (Non-sig)	).02 (-0.26, 0.23), q = 0.944, (Non-sig)
0.06 (-0.32, 0.2), q = 0.831, (Non-sig)	).09 (-0.14, 0.32), q = 0.768, (Non-sig)
0.13 (-0.13, 0.4), q = 0.64, (Non-sig)	).07 (-0.17, 0.31), q = 0.814, (Non-sig)
-0.53 (-0.81, -0.26), q = 0.007, (Sig)	).23 (-0.48, 0.01), q = 0.276, (Non-sig)
.18 (-0.13, 0.49), q = 0.572, (Non-sig)	).16 (-0.12, 0.44), q = 0.589, (Non-sig)
.19 (-0.08, 0.46), q = 0.443, (Non-sig)	0.3 (0.07, 0.54), q = 0.154, (Non-sig)
.19 (-0.08, 0.45), q = 0.451, (Non-sig)	).06 (-0.29, 0.18), q = 0.831, (Non-sig)
-0.03 (-0.3, 0.23), q = 0.9, (Non-sig)	).19 (-0.05, 0.42), q = 0.387, (Non-sig)
.09 (-0.34, 0.17), q = 0.778, (Non-sig)	-0.1 (-0.33, 0.13), q = 0.726, (Non-sig)

Supplementary Table 17. Associations of anthropometric, psychiatric, social, behavioural, so

	Beta (95% Confidence inter	
**PRS predictor**	**Binge eating onset (*n* = 408)**	**Low weight onset (*n* = 587)**
**ADHD**	-0.02 (-1.26, 1.22), q = 0.996, (Non-sig)	0.17 (-1.12, 1.46), q = 0.967, (Non-sig)
**Age at menarche**	1.03 (-0.11, 2.16), q = 0.713, (Non-sig)	0.3 (-0.92, 1.51), q = 0.933, (Non-sig)
**Alcohol dependence**	0.58 (-0.64, 1.81), q = 0.885, (Non-sig)	-1.02 (-2.25, 0.21), q = 0.713, (Non-sig)
**Alcohol use**	0.23 (-0.87, 1.33), q = 0.933, (Non-sig)	0.34 (-0.89, 1.56), q = 0.933, (Non-sig)
**Anorexia nervosa**	-0.54 (-1.66, 0.59), q = 0.885, (Non-sig)	0.27 (-0.98, 1.52), q = 0.933, (Non-sig)
**Anxiety**	0.87 (-0.25, 1.99), q = 0.713, (Non-sig)	-0.44 (-1.68, 0.8), q = 0.918, (Non-sig)
**Autism spectrum disorder**	0.23 (-0.88, 1.35), q = 0.933, (Non-sig)	0.96 (-0.24, 2.16), q = 0.713, (Non-sig)
**BMI**	0 (-1.14, 1.14), q = 0.999, (Non-sig)	-0.92 (-2.09, 0.25), q = 0.713, (Non-sig)
**Bipolar disorder**	0.48 (-0.69, 1.65), q = 0.918, (Non-sig)	-0.09 (-1.32, 1.14), q = 0.994, (Non-sig)
**Cannabis use**	-0.37 (-1.52, 0.78), q = 0.919, (Non-sig)	0.19 (-1.02, 1.4), q = 0.967, (Non-sig)
**Childhood obesity**	-0.29 (-1.53, 0.94), q = 0.933, (Non-sig)	1 (-0.2, 2.2), q = 0.713, (Non-sig)
**Educational attainment**	-0.44 (-1.6, 0.72), q = 0.918, (Non-sig)	0.47 (-0.78, 1.72), q = 0.918, (Non-sig)
**Household income**	0.14 (-1.01, 1.28), q = 0.967, (Non-sig)	-0.72 (-1.92, 0.47), q = 0.885, (Non-sig)
**Insomnia**	0.85 (-0.33, 2.02), q = 0.743, (Non-sig)	-0.17 (-1.32, 0.97), q = 0.967, (Non-sig)
**Major depressive disorder**	0.86 (-0.29, 2.01), q = 0.716, (Non-sig)	-0.98 (-2.22, 0.26), q = 0.713, (Non-sig)
**Neuroticism**	0.46 (-0.67, 1.58), q = 0.918, (Non-sig)	0.48 (-0.74, 1.7), q = 0.918, (Non-sig)
**OCD**	-0.01 (-1.18, 1.16), q = 0.996, (Non-sig)	-0.61 (-1.79, 0.56), q = 0.885, (Non-sig)
**PTSD**	-1.11 (-2.27, 0.05), q = 0.713, (Non-sig)	-0.43 (-1.64, 0.78), q = 0.918, (Non-sig)
**Panic disorder**	0.29 (-0.86, 1.44), q = 0.933, (Non-sig)	-0.26 (-1.52, 0.99), q = 0.933, (Non-sig)
**Risk-taking behaviour**	-0.51 (-1.68, 0.66), q = 0.918, (Non-sig)	-0.45 (-1.77, 0.87), q = 0.918, (Non-sig)
**Schizophrenia**	0.03 (-1.14, 1.2), q = 0.996, (Non-sig)	0.76 (-0.46, 1.98), q = 0.885, (Non-sig)
**Smoking (heavy)**	-0.21 (-1.71, 1.3), q = 0.967, (Non-sig)	-0.53 (-2.01, 0.96), q = 0.918, (Non-sig)
**Smoking (lifetime)**	0.31 (-0.82, 1.44), q = 0.933, (Non-sig)	-0.13 (-1.36, 1.09), q = 0.967, (Non-sig)
**Social deprivation (Townsend**	-0.42 (-1.64, 0.8), q = 0.918, (Non-sig)	-0.29 (-1.5, 0.91), q = 0.933, (Non-sig)
**Tiredness**	-0.86 (-2.08, 0.37), q = 0.784, (Non-sig)	-0.65 (-1.89, 0.59), q = 0.885, (Non-sig)
**Type 2 diabetes**	-0.88 (-2.05, 0.28), q = 0.716, (Non-sig)	-0.14 (-1.33, 1.05), q = 0.967, (Non-sig)

Note. q values are rounded up to 3 decimal points.

matic, personality polygenic risk score associations with age at onset of eating disorder symptoms

rvals), False Discovery Rate *q*-value

**elf-induced vomiting onset (*n* = 12**	**Laxatives onset (*n* = 119)**	**Fasting onset (*n* = 755)**
1.08 (-0.73, 2.9), q = 0.885, (Non-sig)	1.37 (-1.42, 4.17), q = 0.885, (Non-sig)	-0.57 (-1.62, 0.48), q = 0.885, (Non-sig)
1.01 (-0.91, 2.93), q = 0.885, (Non-sig)	-0.26 (-2.99, 2.48), q = 0.988, (Non-sig)	0.98 (-0.05, 2), q = 0.713, (Non-sig)
0.41 (-1.29, 2.1), q = 0.933, (Non-sig)	-0.94 (-3.51, 1.64), q = 0.918, (Non-sig)	-0.06 (-1.05, 0.93), q = 0.994, (Non-sig)
-0.05 (-1.72, 1.63), q = 0.996, (Non-sig)	-0.29 (-2.75, 2.18), q = 0.967, (Non-sig)	-0.13 (-1.16, 0.9), q = 0.967, (Non-sig)
-0.83 (-2.46, 0.79), q = 0.885, (Non-sig)	3.89 (-6.58, -1.19), q = 0.448, (Non-sig)	-0.01 (-1.04, 1.02), q = 0.996, (Non-sig)
-0.43 (-2.11, 1.25), q = 0.933, (Non-sig)	-0.07 (-2.8, 2.65), q = 0.996, (Non-sig)	-1.26 (-2.32, -0.2), q = 0.712, (Non-sig)
-0.6 (-2.32, 1.12), q = 0.918, (Non-sig)	1.72 (-0.82, 4.25), q = 0.817, (Non-sig)	-0.84 (-1.89, 0.22), q = 0.713, (Non-sig)
1.04 (-0.73, 2.82), q = 0.885, (Non-sig)	2.67 (0.01, 5.33), q = 0.713, (Non-sig)	-0.36 (-1.38, 0.66), q = 0.918, (Non-sig)
-0.97 (-2.75, 0.81), q = 0.885, (Non-sig)	-1.84 (-4.52, 0.84), q = 0.808, (Non-sig)	-0.25 (-1.29, 0.79), q = 0.933, (Non-sig)
0.51 (-1.23, 2.24), q = 0.933, (Non-sig)	3.07 (0.41, 5.72), q = 0.712, (Non-sig)	-0.13 (-1.15, 0.89), q = 0.967, (Non-sig)
-0.25 (-1.94, 1.44), q = 0.967, (Non-sig)	2.27 (-0.51, 5.04), q = 0.713, (Non-sig)	-0.83 (-1.83, 0.18), q = 0.713, (Non-sig)
-0.88 (-2.79, 1.02), q = 0.888, (Non-sig)	-0.99 (-3.91, 1.92), q = 0.918, (Non-sig)	0.57 (-0.43, 1.56), q = 0.885, (Non-sig)
1.41 (-0.24, 3.06), q = 0.713, (Non-sig)	-0.36 (-3.23, 2.5), q = 0.967, (Non-sig)	0.9 (-0.11, 1.91), q = 0.713, (Non-sig)
0.31 (-1.57, 2.19), q = 0.967, (Non-sig)	1.59 (-1.14, 4.32), q = 0.885, (Non-sig)	-0.06 (-1.08, 0.95), q = 0.994, (Non-sig)
0.84 (-0.91, 2.58), q = 0.885, (Non-sig)	-0.7 (-3.27, 1.87), q = 0.933, (Non-sig)	0.22 (-0.83, 1.27), q = 0.933, (Non-sig)
-0.05 (-1.74, 1.64), q = 0.996, (Non-sig)	0.21 (-2.42, 2.85), q = 0.994, (Non-sig)	-0.78 (-1.77, 0.21), q = 0.713, (Non-sig)
-1.19 (-3.04, 0.66), q = 0.859, (Non-sig)	3.03 (-5.68, -0.38), q = 0.712, (Non-sig)	-0.39 (-1.43, 0.66), q = 0.918, (Non-sig)
0.9 (-1.02, 2.82), q = 0.888, (Non-sig)	-1.8 (-4.23, 0.64), q = 0.737, (Non-sig)	-0.15 (-1.21, 0.91), q = 0.967, (Non-sig)
0.55 (-1.53, 2.63), q = 0.933, (Non-sig)	1 (-1.97, 3.97), q = 0.918, (Non-sig)	0.38 (-0.66, 1.43), q = 0.918, (Non-sig)
0.03 (-1.8, 1.87), q = 0.996, (Non-sig)	0.65 (-1.95, 3.25), q = 0.933, (Non-sig)	0.89 (-0.17, 1.95), q = 0.713, (Non-sig)
1.02 (-0.92, 2.96), q = 0.885, (Non-sig)	0.05 (-2.77, 2.87), q = 0.996, (Non-sig)	0.31 (-0.73, 1.35), q = 0.933, (Non-sig)
-0.07 (-2.08, 1.95), q = 0.996, (Non-sig)	-0.01 (-3.55, 3.53), q = 0.999, (Non-sig)	0.26 (-0.96, 1.49), q = 0.933, (Non-sig)
1.47 (-0.32, 3.26), q = 0.713, (Non-sig)	-0.24 (-3.23, 2.75), q = 0.994, (Non-sig)	0.51 (-0.51, 1.53), q = 0.885, (Non-sig)
-1.08 (-3.05, 0.89), q = 0.885, (Non-sig)	0.36 (-2.88, 3.61), q = 0.967, (Non-sig)	-0.88 (-1.9, 0.14), q = 0.713, (Non-sig)
0.65 (-1.12, 2.42), q = 0.918, (Non-sig)	-1.24 (-4.2, 1.72), q = 0.918, (Non-sig)	0.03 (-1.03, 1.09), q = 0.996, (Non-sig)
-0.23 (-2.16, 1.71), q = 0.967, (Non-sig)	-1.49 (-4.41, 1.43), q = 0.885, (Non-sig)	-0.4 (-1.44, 0.63), q = 0.918, (Non-sig)

in males.

Excessive exercise onset (*n* = 526)
0.08 (-0.98, 1.13), q = 0.994, (Non-sig)
1.71 (0.7, 2.71), q = 0.145, (Non-sig)
0.24 (-0.78, 1.26), q = 0.933, (Non-sig)
-0.79 (-1.8, 0.23), q = 0.713, (Non-sig)
-0.88 (-1.85, 0.08), q = 0.713, (Non-sig)
-0.34 (-1.36, 0.69), q = 0.919, (Non-sig)
0.68 (-0.37, 1.73), q = 0.859, (Non-sig)
-0.8 (-1.85, 0.24), q = 0.713, (Non-sig)
-0.17 (-1.23, 0.89), q = 0.967, (Non-sig)
0.24 (-0.75, 1.22), q = 0.933, (Non-sig)
0.13 (-0.88, 1.15), q = 0.967, (Non-sig)
-0.47 (-1.51, 0.56), q = 0.892, (Non-sig)
-0.34 (-1.35, 0.67), q = 0.918, (Non-sig)
0.51 (-0.55, 1.56), q = 0.885, (Non-sig)
0.31 (-0.73, 1.35), q = 0.933, (Non-sig)
-0.57 (-1.58, 0.45), q = 0.885, (Non-sig)
-1.18 (-2.18, -0.17), q = 0.712, (Non-sig)
-1.13 (-2.22, -0.04), q = 0.713, (Non-sig)
0.25 (-0.82, 1.32), q = 0.933, (Non-sig)
0.52 (-0.51, 1.55), q = 0.885, (Non-sig)
0.33 (-0.74, 1.39), q = 0.933, (Non-sig)
0.09 (-1.19, 1.36), q = 0.994, (Non-sig)
0.17 (-0.88, 1.22), q = 0.967, (Non-sig)
-0.03 (-1.11, 1.05), q = 0.996, (Non-sig)
0.51 (-0.54, 1.56), q = 0.885, (Non-sig)
-0.19 (-1.24, 0.87), q = 0.967, (Non-sig)

Supplementary Table 18. Associations of anthropometric, psychiatric, social, behavioural, somatic, pe

PRS predictor	Odds ratio (95% Confidence intervals), False Discover	
	l of weight gain (cases *n* = 3,578; control *n* = 1	low weight (cases *n* = 3,153; contro
**ADHD**	1.01 (0.93, 1.09), q = 0.948, (Non-sig)	1.06 (0.99, 1.14), q = 0.359, (Non-sig)
**Age at menarche**	0.88 (0.81, 0.95), q = 0.016, (Non-sig)	0.89 (0.83, 0.96), q = 0.021, (Non-sig)
**Alcohol dependence**	1.01 (0.94, 1.1), q = 0.938, (Non-sig)	1 (0.93, 1.07), q = 0.948, (Non-sig)
**Alcohol use**	0.99 (0.92, 1.07), q = 0.948, (Non-sig)	1 (0.93, 1.07), q = 0.948, (Non-sig)
**Anorexia nervosa**	1.18 (1.09, 1.28), q = 0.001, (Sig)	1.06 (0.99, 1.13), q = 0.402, (Non-sig)
**Anxiety**	1.01 (0.93, 1.09), q = 0.948, (Non-sig)	1.04 (0.96, 1.12), q = 0.655, (Non-sig)
**Autism spectrum disorder**	0.93 (0.86, 1), q = 0.298, (Non-sig)	0.97 (0.9, 1.04), q = 0.655, (Non-sig)
**BMI**	1.25 (1.15, 1.36), q = 0, (Sig)	1.26 (1.18, 1.36), q = 0, (Sig)
**Bipolar disorder**	1.06 (0.98, 1.15), q = 0.415, (Non-sig)	1.08 (1, 1.16), q = 0.222, (Non-sig)
**Cannabis use**	1.08 (1, 1.17), q = 0.302, (Non-sig)	1.08 (1.01, 1.16), q = 0.189, (Non-sig)
**Childhood obesity**	1.25 (1.16, 1.35), q = 0, (Sig)	1.18 (1.1, 1.26), q = 0, (Sig)
**Educational attainment**	0.99 (0.91, 1.07), q = 0.948, (Non-sig)	0.93 (0.86, 0.99), q = 0.217, (Non-sig)
**Household income**	1.02 (0.95, 1.11), q = 0.908, (Non-sig)	0.98 (0.92, 1.05), q = 0.91, (Non-sig)
**Insomnia**	0.99 (0.91, 1.07), q = 0.938, (Non-sig)	1.08 (1.01, 1.16), q = 0.196, (Non-sig)
**Major depressive disorder**	1.03 (0.95, 1.12), q = 0.739, (Non-sig)	1.12 (1.04, 1.21), q = 0.021, (Non-sig)
**Neuroticism**	1.02 (0.94, 1.1), q = 0.938, (Non-sig)	1.03 (0.96, 1.11), q = 0.73, (Non-sig)
**OCD**	0.95 (0.88, 1.03), q = 0.513, (Non-sig)	0.95 (0.89, 1.02), q = 0.463, (Non-sig)
**PTSD**	1.01 (0.93, 1.1), q = 0.938, (Non-sig)	1.05 (0.98, 1.13), q = 0.416, (Non-sig)
**Panic disorder**	0.95 (0.87, 1.03), q = 0.463, (Non-sig)	0.96 (0.89, 1.03), q = 0.513, (Non-sig)
**Risk-taking behaviour**	1.03 (0.95, 1.11), q = 0.869, (Non-sig)	1.12 (1.04, 1.2), q = 0.021, (Non-sig)
**Schizophrenia**	1.01 (0.93, 1.1), q = 0.948, (Non-sig)	0.99 (0.92, 1.06), q = 0.948, (Non-sig)
**Smoking (heavy)**	1 (0.91, 1.1), q = 0.948, (Non-sig)	1.04 (0.95, 1.13), q = 0.73, (Non-sig)
**Smoking (lifetime)**	1.04 (0.96, 1.12), q = 0.73, (Non-sig)	1.11 (1.03, 1.19), q = 0.033, (Non-sig)
**Social deprivation (Townsend)**	1.02 (0.95, 1.11), q = 0.908, (Non-sig)	1.01 (0.95, 1.09), q = 0.938, (Non-sig)
**Tiredness**	0.98 (0.91, 1.07), q = 0.938, (Non-sig)	1.01 (0.94, 1.08), q = 0.948, (Non-sig)
**Type 2 diabetes**	1.05 (0.97, 1.14), q = 0.513, (Non-sig)	1.06 (0.99, 1.14), q = 0.402, (Non-sig)

Note. q values are rounded up to 3 decimal points.

ersonality polygenic risk score associations with cognitive sympto

ry Rate *q*-value	
**Self-worth dependent body**	**Self-worth dependent body**
0.98 (0.89, 1.07), q = 0.908, (Non-sig)	0.96 (0.82, 1.13), q = 0.91, (Non-sig)
0.91 (0.84, 1), q = 0.259, (Non-sig)	1.13 (0.96, 1.33), q = 0.402, (Non-sig)
1.08 (0.99, 1.18), q = 0.385, (Non-sig)	0.98 (0.83, 1.15), q = 0.948, (Non-sig)
0.98 (0.9, 1.07), q = 0.938, (Non-sig)	1.13 (0.96, 1.33), q = 0.402, (Non-sig)
1.09 (1, 1.19), q = 0.259, (Non-sig)	1.14 (0.97, 1.34), q = 0.402, (Non-sig)
1.06 (0.96, 1.16), q = 0.53, (Non-sig)	1.13 (0.96, 1.34), q = 0.402, (Non-sig)
0.95 (0.87, 1.03), q = 0.513, (Non-sig)	1.01 (0.86, 1.19), q = 0.948, (Non-sig)
1.15 (1.05, 1.26), q = 0.03, (Non-sig)	0.86 (0.73, 1.01), q = 0.317, (Non-sig)
1.11 (1.02, 1.21), q = 0.16, (Non-sig)	1.01 (0.86, 1.18), q = 0.948, (Non-sig)
1.04 (0.95, 1.14), q = 0.73, (Non-sig)	1.01 (0.86, 1.19), q = 0.948, (Non-sig)
1.15 (1.06, 1.25), q = 0.019, (Non-sig)	1.01 (0.86, 1.18), q = 0.948, (Non-sig)
1.07 (0.98, 1.17), q = 0.415, (Non-sig)	1.25 (1.06, 1.47), q = 0.07, (Non-sig)
0.98 (0.89, 1.07), q = 0.91, (Non-sig)	1.15 (0.98, 1.34), q = 0.372, (Non-sig)
1.02 (0.93, 1.11), q = 0.938, (Non-sig)	1.01 (0.86, 1.19), q = 0.948, (Non-sig)
1.07 (0.98, 1.18), q = 0.402, (Non-sig)	1.02 (0.87, 1.21), q = 0.948, (Non-sig)
1.03 (0.94, 1.12), q = 0.908, (Non-sig)	0.98 (0.83, 1.15), q = 0.948, (Non-sig)
1.05 (0.96, 1.15), q = 0.632, (Non-sig)	0.97 (0.83, 1.14), q = 0.938, (Non-sig)
1.05 (0.96, 1.15), q = 0.644, (Non-sig)	0.95 (0.8, 1.12), q = 0.869, (Non-sig)
0.93 (0.85, 1.02), q = 0.402, (Non-sig)	1.11 (0.95, 1.3), q = 0.513, (Non-sig)
1.03 (0.94, 1.12), q = 0.889, (Non-sig)	1.07 (0.91, 1.25), q = 0.742, (Non-sig)
1.01 (0.92, 1.11), q = 0.948, (Non-sig)	0.93 (0.79, 1.09), q = 0.73, (Non-sig)
1.05 (0.94, 1.16), q = 0.73, (Non-sig)	1.01 (0.84, 1.22), q = 0.948, (Non-sig)
1.01 (0.92, 1.1), q = 0.948, (Non-sig)	1.03 (0.88, 1.22), q = 0.938, (Non-sig)
1 (0.92, 1.1), q = 0.948, (Non-sig)	1.07 (0.91, 1.26), q = 0.73, (Non-sig)
0.98 (0.9, 1.07), q = 0.938, (Non-sig)	1.05 (0.89, 1.23), q = 0.908, (Non-sig)
1.07 (0.98, 1.17), q = 0.416, (Non-sig)	0.88 (0.76, 1.03), q = 0.402, (Non-sig)

Supplementary Table 19. Associations of anthropometric, psychiatric, social, behavioural, so

PRS predictor	Odds ratio (95% Confiden	
	Afraid of weight gain (*n* = 3,633)	Feel fat at low weight (*n* = 3,209)
**ADHD**	1.03 (0.97, 1.11), q = 0.526, (Non-sig)	1.12 (1.05, 1.2), q = 0.006, (Sig)
**Age at menarche**	0.96 (0.9, 1.03), q = 0.449, (Non-sig)	0.96 (0.9, 1.02), q = 0.438, (Non-sig)
**Alcohol dependence**	0.98 (0.92, 1.05), q = 0.803, (Non-sig)	0.99 (0.93, 1.05), q = 0.827, (Non-sig)
**Alcohol use**	1.04 (0.97, 1.11), q = 0.526, (Non-sig)	0.96 (0.9, 1.02), q = 0.437, (Non-sig)
**Anorexia nervosa**	1.07 (1, 1.14), q = 0.195, (Non-sig)	1.01 (0.95, 1.08), q = 0.811, (Non-sig)
**Anxiety**	0.99 (0.92, 1.06), q = 0.822, (Non-sig)	0.97 (0.91, 1.04), q = 0.569, (Non-sig)
**Autism spectrum disorder**	0.97 (0.91, 1.03), q = 0.554, (Non-sig)	1.02 (0.96, 1.09), q = 0.688, (Non-sig)
**BMI**	1.1 (1.03, 1.18), q = 0.047, (Non-sig)	1.18 (1.1, 1.26), q = 0, (Sig)
**Bipolar disorder**	0.97 (0.9, 1.03), q = 0.526, (Non-sig)	0.93 (0.87, 0.99), q = 0.12, (Non-sig)
**Cannabis use**	1.03 (0.97, 1.1), q = 0.527, (Non-sig)	1.1 (1.03, 1.17), q = 0.042, (Non-sig)
**Childhood obesity**	1.08 (1.01, 1.15), q = 0.113, (Non-sig)	1.03 (0.97, 1.1), q = 0.569, (Non-sig)
**Educational attainment**	0.96 (0.9, 1.03), q = 0.476, (Non-sig)	0.9 (0.84, 0.96), q = 0.014, (Non-sig)
**Household income**	1.01 (0.94, 1.08), q = 0.95, (Non-sig)	0.98 (0.92, 1.04), q = 0.7, (Non-sig)
**Insomnia**	1.09 (1.02, 1.17), q = 0.054, (Non-sig)	1.07 (1, 1.14), q = 0.213, (Non-sig)
**Major depressive disorder**	1.05 (0.98, 1.13), q = 0.344, (Non-sig)	1.06 (0.99, 1.13), q = 0.26, (Non-sig)
**Neuroticism**	1 (0.94, 1.07), q = 0.958, (Non-sig)	0.95 (0.89, 1.01), q = 0.263, (Non-sig)
**OCD**	1.01 (0.94, 1.08), q = 0.952, (Non-sig)	0.99 (0.93, 1.05), q = 0.865, (Non-sig)
**PTSD**	1.03 (0.96, 1.1), q = 0.669, (Non-sig)	1.05 (0.99, 1.13), q = 0.315, (Non-sig)
**Panic disorder**	0.94 (0.88, 1), q = 0.232, (Non-sig)	0.99 (0.93, 1.06), q = 0.883, (Non-sig)
**Risk-taking behaviour**	1.02 (0.95, 1.09), q = 0.71, (Non-sig)	1.04 (0.97, 1.11), q = 0.479, (Non-sig)
**Schizophrenia**	1.02 (0.95, 1.09), q = 0.8, (Non-sig)	0.96 (0.9, 1.03), q = 0.517, (Non-sig)
**Smoking (heavy)**	1.04 (0.96, 1.13), q = 0.517, (Non-sig)	1.07 (0.99, 1.16), q = 0.249, (Non-sig)
**Smoking (lifetime)**	1.03 (0.96, 1.1), q = 0.671, (Non-sig)	1.03 (0.96, 1.09), q = 0.651, (Non-sig)
**Social deprivation (Townsend**	0.98 (0.92, 1.05), q = 0.7, (Non-sig)	1.07 (1.01, 1.14), q = 0.154, (Non-sig)
**Tiredness**	1 (0.94, 1.07), q = 0.958, (Non-sig)	1 (0.94, 1.07), q = 0.958, (Non-sig)
**Type 2 diabetes**	1 (0.93, 1.07), q = 0.958, (Non-sig)	1.03 (0.97, 1.1), q = 0.575, (Non-sig)

Note. q values are rounded up to 3 decimal points.

matic, personality polygenic risk score associations with severity of cognitive

ıce intervals), False Discovery Rate *q*-value

Feel body is larger at low weight (*n* =	Self-worth dependent body
1.01 (0.95, 1.07), q = 0.811, (Non-sig)	1.08 (1.02, 1.14), q = 0.08, (Non-sig)
0.98 (0.92, 1.04), q = 0.7, (Non-sig)	0.95 (0.9, 1.01), q = 0.268, (Non-sig)
1.02 (0.96, 1.09), q = 0.648, (Non-sig)	0.99 (0.93, 1.05), q = 0.86, (Non-sig)
0.98 (0.92, 1.04), q = 0.651, (Non-sig)	1 (0.95, 1.06), q = 0.958, (Non-sig)
1.1 (1.04, 1.17), q = 0.014, (Non-sig)	1.06 (1, 1.12), q = 0.195, (Non-sig)
1.03 (0.97, 1.1), q = 0.526, (Non-sig)	1.05 (0.98, 1.11), q = 0.349, (Non-sig)
0.9 (0.85, 0.96), q = 0.013, (Non-sig)	0.95 (0.9, 1.01), q = 0.26, (Non-sig)
1.12 (1.06, 1.2), q = 0.004, (Sig)	1.16 (1.09, 1.23), q = 0, (Sig)
1.07 (1, 1.13), q = 0.164, (Non-sig)	1 (0.95, 1.06), q = 0.958, (Non-sig)
1.04 (0.98, 1.1), q = 0.424, (Non-sig)	1.08 (1.02, 1.15), q = 0.054, (Non-sig)
1.14 (1.07, 1.21), q = 0.001, (Sig)	1.08 (1.02, 1.15), q = 0.047, (Non-sig)
0.93 (0.88, 0.99), q = 0.129, (Non-sig)	0.88 (0.83, 0.94), q = 0.001, (Sig)
1 (0.94, 1.06), q = 0.958, (Non-sig)	0.98 (0.93, 1.04), q = 0.7, (Non-sig)
1.03 (0.97, 1.09), q = 0.551, (Non-sig)	1.1 (1.04, 1.17), q = 0.014, (Non-sig)
1.02 (0.96, 1.09), q = 0.648, (Non-sig)	1.08 (1.02, 1.15), q = 0.054, (Non-sig)
1.02 (0.96, 1.08), q = 0.7, (Non-sig)	1.04 (0.98, 1.1), q = 0.402, (Non-sig)
0.99 (0.94, 1.05), q = 0.928, (Non-sig)	1.01 (0.95, 1.06), q = 0.956, (Non-sig)
1 (0.94, 1.06), q = 0.958, (Non-sig)	1.06 (1, 1.13), q = 0.195, (Non-sig)
0.98 (0.93, 1.05), q = 0.771, (Non-sig)	1 (0.95, 1.06), q = 0.971, (Non-sig)
1.02 (0.96, 1.08), q = 0.7, (Non-sig)	1.03 (0.98, 1.09), q = 0.49, (Non-sig)
1.02 (0.95, 1.08), q = 0.794, (Non-sig)	1.06 (1, 1.12), q = 0.234, (Non-sig)
1.07 (1, 1.15), q = 0.234, (Non-sig)	1.06 (0.99, 1.14), q = 0.249, (Non-sig)
1.04 (0.97, 1.1), q = 0.483, (Non-sig)	1.13 (1.06, 1.2), q = 0.002, (Sig)
1 (0.94, 1.06), q = 0.958, (Non-sig)	1.04 (0.98, 1.1), q = 0.425, (Non-sig)
1.05 (0.99, 1.11), q = 0.34, (Non-sig)	1.06 (1, 1.12), q = 0.22, (Non-sig)
1.03 (0.97, 1.09), q = 0.582, (Non-sig)	1.05 (0.99, 1.11), q = 0.34, (Non-sig)

: symptoms in females.

Self-worth dependent body
1.09 (1.03, 1.16), q = 0.02, (Non-sig)
0.98 (0.93, 1.04), q = 0.7, (Non-sig)
1 (0.94, 1.06), q = 0.958, (Non-sig)
1 (0.95, 1.06), q = 0.991, (Non-sig)
1.07 (1.01, 1.13), q = 0.108, (Non-sig)
1.03 (0.97, 1.09), q = 0.563, (Non-sig)
1 (0.95, 1.06), q = 0.958, (Non-sig)
1.05 (1, 1.11), q = 0.232, (Non-sig)
0.95 (0.9, 1.01), q = 0.26, (Non-sig)
1.04 (0.98, 1.1), q = 0.438, (Non-sig)
1.08 (1.02, 1.14), q = 0.054, (Non-sig)
0.89 (0.84, 0.94), q = 0.002, (Sig)
0.93 (0.88, 0.98), q = 0.069, (Non-sig)
1.05 (0.99, 1.11), q = 0.265, (Non-sig)
1.05 (0.99, 1.11), q = 0.34, (Non-sig)
1.05 (0.99, 1.11), q = 0.277, (Non-sig)
1.05 (0.99, 1.11), q = 0.271, (Non-sig)
1.04 (0.98, 1.1), q = 0.449, (Non-sig)
0.96 (0.91, 1.02), q = 0.372, (Non-sig)
0.96 (0.91, 1.02), q = 0.397, (Non-sig)
1.04 (0.98, 1.1), q = 0.41, (Non-sig)
1.06 (0.99, 1.13), q = 0.278, (Non-sig)
1.04 (0.98, 1.1), q = 0.433, (Non-sig)
1.04 (0.98, 1.1), q = 0.449, (Non-sig)
1.03 (0.97, 1.09), q = 0.569, (Non-sig)
1.02 (0.96, 1.08), q = 0.7, (Non-sig)

Supplementary Table 20. Associations of anthropometric, psychiatric

PRS predictor	Odds ratio (95% Confidence intervals), False Disco<br>ıid of weight gain (cases *n* = 443; control *n* = 1
**ADHD**	1.03 (0.83, 1.29), q = 0.845, (Non-sig)
**Age at menarche**	0.86 (0.7, 1.05), q = 0.775, (Non-sig)
**Alcohol dependence**	0.95 (0.77, 1.17), q = 0.845, (Non-sig)
**Alcohol use**	0.97 (0.79, 1.19), q = 0.845, (Non-sig)
**Anorexia nervosa**	1.14 (0.92, 1.41), q = 0.834, (Non-sig)
**Anxiety**	0.94 (0.76, 1.16), q = 0.845, (Non-sig)
**Autism spectrum disorder**	0.88 (0.71, 1.08), q = 0.778, (Non-sig)
**BMI**	1.41 (1.16, 1.73), q = 0.061, (Non-sig)
**Bipolar disorder**	0.86 (0.7, 1.06), q = 0.778, (Non-sig)
**Cannabis use**	1.05 (0.85, 1.29), q = 0.845, (Non-sig)
**Childhood obesity**	1.23 (0.99, 1.52), q = 0.775, (Non-sig)
**Educational attainment**	0.94 (0.75, 1.16), q = 0.845, (Non-sig)
**Household income**	0.91 (0.74, 1.11), q = 0.845, (Non-sig)
**Insomnia**	1.08 (0.89, 1.32), q = 0.845, (Non-sig)
**Major depressive disorder**	0.95 (0.77, 1.18), q = 0.845, (Non-sig)
**Neuroticism**	0.85 (0.69, 1.05), q = 0.775, (Non-sig)
**OCD**	1.06 (0.87, 1.3), q = 0.845, (Non-sig)
**PTSD**	0.95 (0.77, 1.18), q = 0.845, (Non-sig)
**Panic disorder**	1.16 (0.93, 1.44), q = 0.778, (Non-sig)
**Risk-taking behaviour**	0.97 (0.77, 1.22), q = 0.845, (Non-sig)
**Schizophrenia**	0.93 (0.76, 1.15), q = 0.845, (Non-sig)
**Smoking (heavy)**	1.1 (0.85, 1.42), q = 0.845, (Non-sig)
**Smoking (lifetime)**	0.97 (0.79, 1.19), q = 0.845, (Non-sig)
**Social deprivation (Townsend)**	1.06 (0.87, 1.3), q = 0.845, (Non-sig)
**Tiredness**	1.1 (0.89, 1.36), q = 0.845, (Non-sig)
**Type 2 diabetes**	1.18 (0.96, 1.45), q = 0.775, (Non-sig)

Note. 'Self-worth dependent on body weight/shape when binge eating' not

c, social, behavioural, somatic, personality polygenic risk score

ɿvery Rate *q*-value	
**t low weight (cases *n* = 488; contro**	**Self-worth dependent body**
1.06 (0.83, 1.36), q = 0.845, (Non-sig)	1.03 (0.85, 1.25), q = 0.845, (Non-sig)
0.98 (0.78, 1.23), q = 0.915, (Non-sig)	0.99 (0.83, 1.18), q = 0.927, (Non-sig)
1.05 (0.83, 1.32), q = 0.845, (Non-sig)	0.91 (0.76, 1.1), q = 0.845, (Non-sig)
0.94 (0.75, 1.19), q = 0.845, (Non-sig)	0.99 (0.83, 1.19), q = 0.927, (Non-sig)
1.22 (0.96, 1.55), q = 0.775, (Non-sig)	0.91 (0.75, 1.1), q = 0.845, (Non-sig)
1.04 (0.83, 1.32), q = 0.845, (Non-sig)	1.08 (0.9, 1.3), q = 0.845, (Non-sig)
1.06 (0.85, 1.32), q = 0.845, (Non-sig)	0.92 (0.77, 1.1), q = 0.845, (Non-sig)
1.23 (0.99, 1.54), q = 0.775, (Non-sig)	1.19 (1, 1.42), q = 0.775, (Non-sig)
0.9 (0.72, 1.13), q = 0.845, (Non-sig)	0.98 (0.82, 1.18), q = 0.915, (Non-sig)
1.13 (0.9, 1.41), q = 0.845, (Non-sig)	0.95 (0.79, 1.14), q = 0.845, (Non-sig)
1.2 (0.95, 1.53), q = 0.775, (Non-sig)	1.04 (0.87, 1.24), q = 0.845, (Non-sig)
1.07 (0.84, 1.36), q = 0.845, (Non-sig)	0.98 (0.81, 1.18), q = 0.845, (Non-sig)
0.82 (0.66, 1.03), q = 0.775, (Non-sig)	1.03 (0.86, 1.23), q = 0.845, (Non-sig)
1.07 (0.86, 1.33), q = 0.845, (Non-sig)	0.97 (0.82, 1.15), q = 0.845, (Non-sig)
1.13 (0.9, 1.43), q = 0.845, (Non-sig)	0.95 (0.79, 1.14), q = 0.845, (Non-sig)
0.82 (0.64, 1.04), q = 0.775, (Non-sig)	0.82 (0.68, 0.99), q = 0.775, (Non-sig)
1.12 (0.9, 1.39), q = 0.845, (Non-sig)	0.91 (0.76, 1.08), q = 0.845, (Non-sig)
0.93 (0.74, 1.18), q = 0.845, (Non-sig)	0.95 (0.79, 1.14), q = 0.845, (Non-sig)
0.95 (0.75, 1.21), q = 0.845, (Non-sig)	1.04 (0.86, 1.25), q = 0.845, (Non-sig)
0.94 (0.73, 1.21), q = 0.845, (Non-sig)	1.13 (0.93, 1.38), q = 0.778, (Non-sig)
1.12 (0.89, 1.41), q = 0.845, (Non-sig)	1.1 (0.92, 1.32), q = 0.845, (Non-sig)
1.12 (0.84, 1.49), q = 0.845, (Non-sig)	1.22 (0.98, 1.53), q = 0.775, (Non-sig)
0.95 (0.76, 1.19), q = 0.845, (Non-sig)	1.01 (0.85, 1.21), q = 0.917, (Non-sig)
1.16 (0.93, 1.44), q = 0.778, (Non-sig)	1.29 (1.08, 1.54), q = 0.206, (Non-sig)
1.17 (0.92, 1.49), q = 0.778, (Non-sig)	1.06 (0.88, 1.27), q = 0.845, (Non-sig)
0.94 (0.75, 1.18), q = 0.845, (Non-sig)	1.12 (0.94, 1.34), q = 0.778, (Non-sig)

t displayed as there were too few controls (n = 50). q values are

Supplementary Table 21. Associations of anthropometric, psychiatric, social, behavioural, so

	**Odds ratio (95% Confidence intervals), Fa**	
**PRS predictor**	**Afraid of weight gain (n = 172)**	**Feel fat at low weight (*n* = 124)**
**ADHD**	1.11 (0.78, 1.58), q = 0.791, (Non-sig)	1.41 (0.86, 2.31), q = 0.574, (Non-sig)
**Age at menarche**	1.18 (0.87, 1.61), q = 0.675, (Non-sig)	0.87 (0.61, 1.23), q = 0.721, (Non-sig)
**Alcohol dependence**	0.87 (0.64, 1.2), q = 0.718, (Non-sig)	1.13 (0.75, 1.68), q = 0.791, (Non-sig)
**Alcohol use**	0.69 (0.5, 0.96), q = 0.439, (Non-sig)	0.79 (0.55, 1.16), q = 0.651, (Non-sig)
**Anorexia nervosa**	1.2 (0.9, 1.6), q = 0.62, (Non-sig)	1.95 (1.28, 2.96), q = 0.115, (Non-sig)
**Anxiety**	1.09 (0.79, 1.51), q = 0.794, (Non-sig)	1.51 (1, 2.28), q = 0.454, (Non-sig)
**Autism spectrum disorder**	1.73 (1.27, 2.37), q = 0.075, (Non-sig)	1.41 (0.98, 2.02), q = 0.454, (Non-sig)
**BMI**	1.01 (0.74, 1.38), q = 0.976, (Non-sig)	1.03 (0.7, 1.52), q = 0.943, (Non-sig)
**Bipolar disorder**	1.38 (1.02, 1.87), q = 0.454, (Non-sig)	1.64 (1.12, 2.39), q = 0.295, (Non-sig)
**Cannabis use**	0.85 (0.62, 1.17), q = 0.684, (Non-sig)	0.9 (0.63, 1.27), q = 0.791, (Non-sig)
**Childhood obesity**	0.76 (0.53, 1.09), q = 0.539, (Non-sig)	0.83 (0.53, 1.3), q = 0.718, (Non-sig)
**Educational attainment**	1.08 (0.78, 1.51), q = 0.809, (Non-sig)	0.81 (0.52, 1.26), q = 0.7, (Non-sig)
**Household income**	0.96 (0.71, 1.3), q = 0.878, (Non-sig)	1.13 (0.76, 1.66), q = 0.791, (Non-sig)
**Insomnia**	0.88 (0.67, 1.16), q = 0.7, (Non-sig)	1.24 (0.85, 1.8), q = 0.669, (Non-sig)
**Major depressive disorder**	0.86 (0.63, 1.18), q = 0.7, (Non-sig)	1.29 (0.83, 2.01), q = 0.669, (Non-sig)
**Neuroticism**	1.01 (0.73, 1.39), q = 0.976, (Non-sig)	1.22 (0.79, 1.88), q = 0.7, (Non-sig)
**OCD**	1.22 (0.9, 1.65), q = 0.608, (Non-sig)	1.32 (0.88, 1.98), q = 0.574, (Non-sig)
**PTSD**	0.87 (0.63, 1.2), q = 0.708, (Non-sig)	0.94 (0.62, 1.44), q = 0.88, (Non-sig)
**Panic disorder**	1.28 (0.94, 1.76), q = 0.539, (Non-sig)	1 (0.67, 1.51), q = 0.986, (Non-sig)
**Risk-taking behaviour**	0.91 (0.65, 1.28), q = 0.791, (Non-sig)	1.54 (0.99, 2.38), q = 0.454, (Non-sig)
**Schizophrenia**	1.06 (0.78, 1.45), q = 0.833, (Non-sig)	1.48 (0.98, 2.23), q = 0.454, (Non-sig)
**Smoking (heavy)**	1.08 (0.73, 1.59), q = 0.833, (Non-sig)	1.15 (0.71, 1.87), q = 0.791, (Non-sig)
**Smoking (lifetime)**	0.84 (0.6, 1.18), q = 0.684, (Non-sig)	0.67 (0.45, 0.99), q = 0.454, (Non-sig)
**Social deprivation (Townsend**	1.32 (0.95, 1.83), q = 0.511, (Non-sig)	1.02 (0.71, 1.47), q = 0.975, (Non-sig)
**Tiredness**	0.8 (0.58, 1.11), q = 0.574, (Non-sig)	0.89 (0.58, 1.35), q = 0.791, (Non-sig)
**Type 2 diabetes**	0.8 (0.58, 1.1), q = 0.549, (Non-sig)	1.1 (0.74, 1.64), q = 0.809, (Non-sig)

Note. q values are rounded up to 3 decimal points.

matic, personality polygenic risk score associations with severity of co

lse Discovery Rate *q*-value	
**Self-worth dependent body**	**Self-worth dependent body**
1.06 (0.85, 1.32), q = 0.809, (Non-sig)	1.15 (0.93, 1.42), q = 0.588, (Non-sig)
0.86 (0.69, 1.06), q = 0.549, (Non-sig)	0.81 (0.67, 0.98), q = 0.454, (Non-sig)
0.92 (0.74, 1.13), q = 0.718, (Non-sig)	1.22 (0.99, 1.52), q = 0.454, (Non-sig)
1.06 (0.86, 1.29), q = 0.794, (Non-sig)	0.88 (0.73, 1.05), q = 0.549, (Non-sig)
1.18 (0.94, 1.5), q = 0.549, (Non-sig)	0.99 (0.82, 1.2), q = 0.976, (Non-sig)
0.98 (0.78, 1.22), q = 0.904, (Non-sig)	1.01 (0.83, 1.23), q = 0.975, (Non-sig)
1.18 (0.97, 1.45), q = 0.511, (Non-sig)	1.16 (0.96, 1.4), q = 0.539, (Non-sig)
1.21 (0.99, 1.48), q = 0.454, (Non-sig)	1.16 (0.96, 1.41), q = 0.539, (Non-sig)
0.91 (0.73, 1.13), q = 0.708, (Non-sig)	0.99 (0.81, 1.21), q = 0.976, (Non-sig)
1.14 (0.92, 1.41), q = 0.666, (Non-sig)	1.03 (0.85, 1.25), q = 0.873, (Non-sig)
1.13 (0.91, 1.41), q = 0.672, (Non-sig)	1.09 (0.88, 1.35), q = 0.718, (Non-sig)
0.94 (0.76, 1.17), q = 0.794, (Non-sig)	0.84 (0.69, 1.02), q = 0.499, (Non-sig)
0.85 (0.7, 1.05), q = 0.539, (Non-sig)	0.87 (0.71, 1.05), q = 0.549, (Non-sig)
1.11 (0.9, 1.36), q = 0.684, (Non-sig)	1.12 (0.91, 1.36), q = 0.675, (Non-sig)
1.03 (0.84, 1.28), q = 0.873, (Non-sig)	0.93 (0.77, 1.13), q = 0.759, (Non-sig)
0.9 (0.72, 1.12), q = 0.684, (Non-sig)	0.98 (0.81, 1.18), q = 0.882, (Non-sig)
1.09 (0.9, 1.33), q = 0.7, (Non-sig)	1.14 (0.93, 1.39), q = 0.62, (Non-sig)
0.93 (0.75, 1.14), q = 0.759, (Non-sig)	0.96 (0.79, 1.18), q = 0.833, (Non-sig)
1.11 (0.89, 1.38), q = 0.7, (Non-sig)	1.01 (0.83, 1.22), q = 0.976, (Non-sig)
0.92 (0.74, 1.15), q = 0.75, (Non-sig)	0.82 (0.67, 1), q = 0.454, (Non-sig)
1.05 (0.84, 1.32), q = 0.809, (Non-sig)	1.05 (0.86, 1.28), q = 0.809, (Non-sig)
0.93 (0.71, 1.22), q = 0.794, (Non-sig)	1.08 (0.83, 1.4), q = 0.791, (Non-sig)
0.93 (0.75, 1.17), q = 0.791, (Non-sig)	0.91 (0.75, 1.1), q = 0.684, (Non-sig)
1.04 (0.85, 1.28), q = 0.833, (Non-sig)	0.99 (0.81, 1.22), q = 0.976, (Non-sig)
1.14 (0.91, 1.43), q = 0.669, (Non-sig)	0.92 (0.75, 1.13), q = 0.722, (Non-sig)
1.08 (0.88, 1.32), q = 0.75, (Non-sig)	1.07 (0.87, 1.3), q = 0.791, (Non-sig)

ɔgnitive symptoms in males.

Feel body parts larger than they are
1.42 (1.1, 1.84), q = 0.281, (Non-sig)
0.87 (0.69, 1.1), q = 0.669, (Non-sig)
1.22 (0.96, 1.55), q = 0.511, (Non-sig)
1.05 (0.83, 1.33), q = 0.83, (Non-sig)
1.24 (0.96, 1.6), q = 0.511, (Non-sig)
1.14 (0.9, 1.46), q = 0.675, (Non-sig)
1.08 (0.86, 1.37), q = 0.766, (Non-sig)
1.3 (1.04, 1.62), q = 0.422, (Non-sig)
1.11 (0.88, 1.4), q = 0.718, (Non-sig)
1.24 (0.98, 1.58), q = 0.49, (Non-sig)
1.05 (0.81, 1.35), q = 0.833, (Non-sig)
0.91 (0.7, 1.17), q = 0.733, (Non-sig)
0.83 (0.66, 1.04), q = 0.511, (Non-sig)
1.12 (0.9, 1.4), q = 0.684, (Non-sig)
1.23 (0.97, 1.56), q = 0.511, (Non-sig)
1.05 (0.82, 1.34), q = 0.833, (Non-sig)
0.96 (0.76, 1.2), q = 0.833, (Non-sig)
1.22 (0.96, 1.55), q = 0.511, (Non-sig)
1.09 (0.85, 1.39), q = 0.766, (Non-sig)
1.14 (0.88, 1.47), q = 0.684, (Non-sig)
1.34 (1.05, 1.71), q = 0.393, (Non-sig)
1.04 (0.78, 1.4), q = 0.878, (Non-sig)
1 (0.8, 1.26), q = 0.986, (Non-sig)
1.2 (0.96, 1.51), q = 0.526, (Non-sig)
1.41 (1.09, 1.83), q = 0.281, (Non-sig)
1.11 (0.88, 1.41), q = 0.708, (Non-sig)
